# Characterization of Leukemia-Inducing Genes Using a Proto-Oncogene/Homeobox Gene Retroviral Human cDNA Library in a Mouse *In Vivo* Model

**DOI:** 10.1371/journal.pone.0143240

**Published:** 2015-11-25

**Authors:** Su Hwa Jang, Sohyun Lee, Hee Yong Chung

**Affiliations:** Department of Biomedical Science, Graduate School of Biomedical Science and Engineering, Hanyang University, Seoul, Republic of Korea; B.C. Cancer Agency, CANADA

## Abstract

The purpose of this research is to develop a method to screen a large number of potential driver mutations of acute myeloid leukemia (AML) using a retroviral cDNA library and murine bone marrow transduction-transplantation system. As a proof-of-concept, murine bone marrow (BM) cells were transduced with a retroviral cDNA library encoding well-characterized oncogenes and homeobox genes, and the virus-transduced cells were transplanted into lethally irradiated mice. The proto-oncogenes responsible for leukemia initiation were identified by PCR amplification of cDNA inserts from genomic DNA isolated from leukemic cells. In an initial screen of ten leukemic mice, the *MYC* proto-oncogene was detected in all the leukemic mice. Of ten leukemic mice, 3 (30%) had *MYC* as the only transgene, and seven mice (70%) had additional proto-oncogene inserts. We repeated the same experiment after removing *MYC*-related genes from the library to characterize additional leukemia-inducing gene combinations. Our second screen using the *MYC*-deleted proto-oncogene library confirmed *MEIS1*and the *HOX* family as cooperating oncogenes in leukemia pathogenesis. The model system we introduced in this study will be valuable in functionally screening novel combinations of genes for leukemogenic potential *in vivo*, and the system will help in the discovery of new targets for leukemia therapy.

## Introduction

Leukemia is a disease caused by multiple alterations of cellular proto-oncogenes in leukemic stem cells. For example, in the Eμ -myc mouse, a transgenic animal model of B-cell leukemia, an incubation period of 4 to 6 months is required to accumulate additional genetic alterations for B cell leukemia to appear [1]. In addition, clinical studies have clearly shown that an activating mutation of *PDGFRB*, *RAS* or *KIT* cooperates with an existing *AML-ETO* fusion gene generated by chromosomal translocation in AML patients [2,3]. Current evaluation of potential driver mutations is limited by low-throughput functional approaches. Although several different model systems have been introduced to search for genes responsible for leukemia induction [4], there has never been a systematic exploration for proto-oncogene or combinations of cooperative proto-oncogenes that can induce leukemia.

Here, we describe a system of screening for leukemia-inducing genes or gene combinations in mice using a cDNA library cloned into the MSCV retroviral backbone. The cDNA library was constructed using retroviral vectors to facilitate gene transfer into primary bone marrow cells and to allow elucidation of cDNAs transferred to leukemic cells by PCR amplification of cDNA inserts from the genomic DNA of the leukemic cells. The monoclonal or oligoclonal nature of most leukemias and permanent integration of proviral DNA into host chromosomes upon virus transduction allow identification of cDNAs transferred to leukemic cells. The screening process will provide lists of candidate gene combinations that have leukemogenic potential, and the validation procedure is relatively straightforward. As a test, we constructed a retroviral expression library of proto-oncogenes and homeobox family of transcription factor genes to explore the gene(s) that induce leukemia either alone or in combination in a mouse leukemia model. For this, we transduced mouse whole bone marrow cells with a retroviral cDNA library, and the transduced cells were transplanted into syngenic irradiated mice. After a 6- to 8-week incubation period, all the mice developed leukemia, and the identity of the cDNA present in the leukemic cells was determined by PCR amplification of the viral inserts from the genomic DNA and subsequent DNA sequencing of the amplified cDNA fragments. Surprisingly, the leukemic cells from all ten host mice from the first series of the screen were found to contain *MYC* proto-oncogenes either alone or in combination with secondary proto-oncogenes. All the mice showed an AML-like phenotype, except the mice with *PIM2* as a secondary proto-oncogene, which showed a pro-B-cell phenotype of leukemia as well.

To further characterize the leukemia-initiating gene combinations in our cDNA library, we removed *MYC*-related genes from our library and repeated the experiment. We reasoned that removal of the powerful *MYC* oncogene would reveal the leukemia-initiating potential of additional cDNAs. The second experiment with *MYC*-deleted library showed that members of the homeobox gene family constitute another potent leukemia-inducing gene combination. The Hox family of homeobox genes is crucial in body pattern formation during ontogenesis [5], and the homeobox family genes have been known to be involved in hematopoietic stem cell renewal and differentiation [6]. In addition, many human leukemia samples show elevated levels of homeobox proteins [7]. In this study, all eight mice in the second series of the screen developed myeloid leukemia, and the leukemic cells were shown to have at least one *HOX* family gene along with *MEIS1* as their retroviral inserts. Among the gene combinations, several *HOX* and *MEIS1* gene combinations were newly discovered to have leukemogenic potential.

Since our screening experiments result in combinations of proto-oncogenes already known as leukemia driver genes as well as several novel combinations of proto-oncogenes, our results clearly shows the usefulness of the model system. The model system introduced in this work will be useful in functional analysis of the comprehensive list of genes and gene combinations discovered by recent genome sequencing studies for their leukemogenic potential.

## Materials and Methods

### Construction of retroviral cDNA library of proto-oncogenes and homeoboxgenes

The 176 full-length proto-oncogenes and homeoboxgenes (**S1 Table**, OpenBiosystems, Huntsville, AL) were individually cloned into EcoR I (or Spe I) and Not I restriction enzyme sites of the modified pMSCV retroviral vector (Clontech, PaloAlto, CA, USA). The pMSCV vector was modified to express both full-length cDNA and GFP through the internal ribosomal entry site (IRES). For efficient expression of the cloned gene, the Kozak sequence[8] was inserted at the 5’ of the start codon. The HA (Hemagglutinin)-tag was inserted at the 5’ end of the cloned cDNA sequence to facilitate the detection of protein expression. The list of proto-oncogene and homeobox family genes is provided in the S1 Table. The retroviral cDNA library was constructed by first mixing a constant amount of plasmid DNAs from 176 clones and then producing ecotropic retrovirus with the plasmid mixture.

### Ecotropic retrovirus cocktail production and bone marrow transplantation

The retroviral vector plasmids were transfected into the 293T cells along with ecotropic packaging plasmid pIK6.1MCV.ecopac.UTD (Ecopac: M. Finer Cell Genosys, Redwood City, CA). The viral supernatants were harvested two days after transfection, passed through 0.45-μm filter, and then the frozen aliquots were stored in -80°C. Mononuclear bone marrow cells were harvested from 6- to 10-week-old C57BL/6 or CD45 congenic C57BL/6.SJL (Jackson Laboratory, Barr Harbor, ME) mice treated with 15 mg/kg 5-FU(Fluorouracil) for two days. Cells were stimulated for 24 hrs with cytokine mixtures containing mIL-3 (5 ng/ml; R&D Biosystems), mSCF (50 ng/ml; R&D Biosystems), mFlt-3/flk-2 (50 ng/ml; R&D Biosystems), and mIL-6 (10 ng/ml; R&D Biosystems). Retrovirus transduction was performed twice in the presence of a cytokine mixture and polybrene (4 μg/ml; Sigma, St. Louis, MO) at day 1 and day 2. On day 3, 10^6^ virus-transduced cells were injected intravenously into lethally irradiated (850 rad) recipient mice along with 10^6^ fresh bone marrow cells for radioprotection.

### Analysis of mice

Four- to six-week-old female C57BL/6 and CD45 congenic C57BL/6.SJL mice were purchased from Jackson Laboratory (Bar Harbor, ME). Mice were housed and bred under SPF conditions in the mouse breeding facility of Hanyang University. The use of experimental animals was in accordance with the Hanyang University Institutional Animal Care and Use Committee (IACUC) guidelines. The Hanyang University Institutional Animal Care and Use Committee (IACUC) approved the research proposal related to the present study (Permission No.: HY-IACUC-1-016). The mice were sacrificed by CO_2_ inhalation when necessary. The mice transplanted with retrovirus-transduced bone marrow cells were monitored twice a week for the symptoms of leukemic disease by visual examination. Once the mice start showing signs of less mobility, the mice were monitored twice a day. Moribund mice with ruffled hair, pronounced weight loss, hind limb paralysis and hunched back were sacrificed by CO_2_ inhalation and analyzed for leukemia phenotype. During the two experiments in which the mice with leukemic symptoms were analyzed after euthanization, only one mouse died prior to euthanization. Mononuclear cells from spleen, lymph nodes, and bone marrow were prepared by ACK lysis buffer (0.15 M NH_4_Cl, 1 mM KHCO_3_, 0.1 mM EDTA) and LIF (Low ionic strength) buffer. For immune-phenotyping, 10^6^ mononuclear cells were resuspended in 50 μl staining buffer (PBS with 1% FBS) and incubated on ice for 20 min with appropriate antibodies: Gr-1, PE-B220, MAC-1 (BD Bioscience, San Diego, CA). Cells were analyzed in a FACS Calibur (BD Bioscience, San Diego, CA).

### Identification of cDNAs in proviral sequences of leukemic cells

Genomic DNAs were isolated from mouse spleen, bone marrow, or lymph nodes tissues. And the cDNAs within the proviral sequences integrated into chromosomes of leukemic cells were amplified by PCR with the primers corresponding to the retroviral backbone sequence upstream and downstream of the cDNA insert (forward primer: 5’-tctaggcgccggaattagatcc, reverse primer: 5’-ttattccaagcggcttcggcca). The size of the cDNA fragments amplified by PCR has 173 bp additional sequence which includes HA tag and retroviral backbone sequence. The PCR products were visualized by agarose gel electrophoresis, the DNA fragments from the individual bands were eluted and the identity of the cDNA was determined by nucleotide sequencing.

## Results

### Experimental design

**Fig 1:**
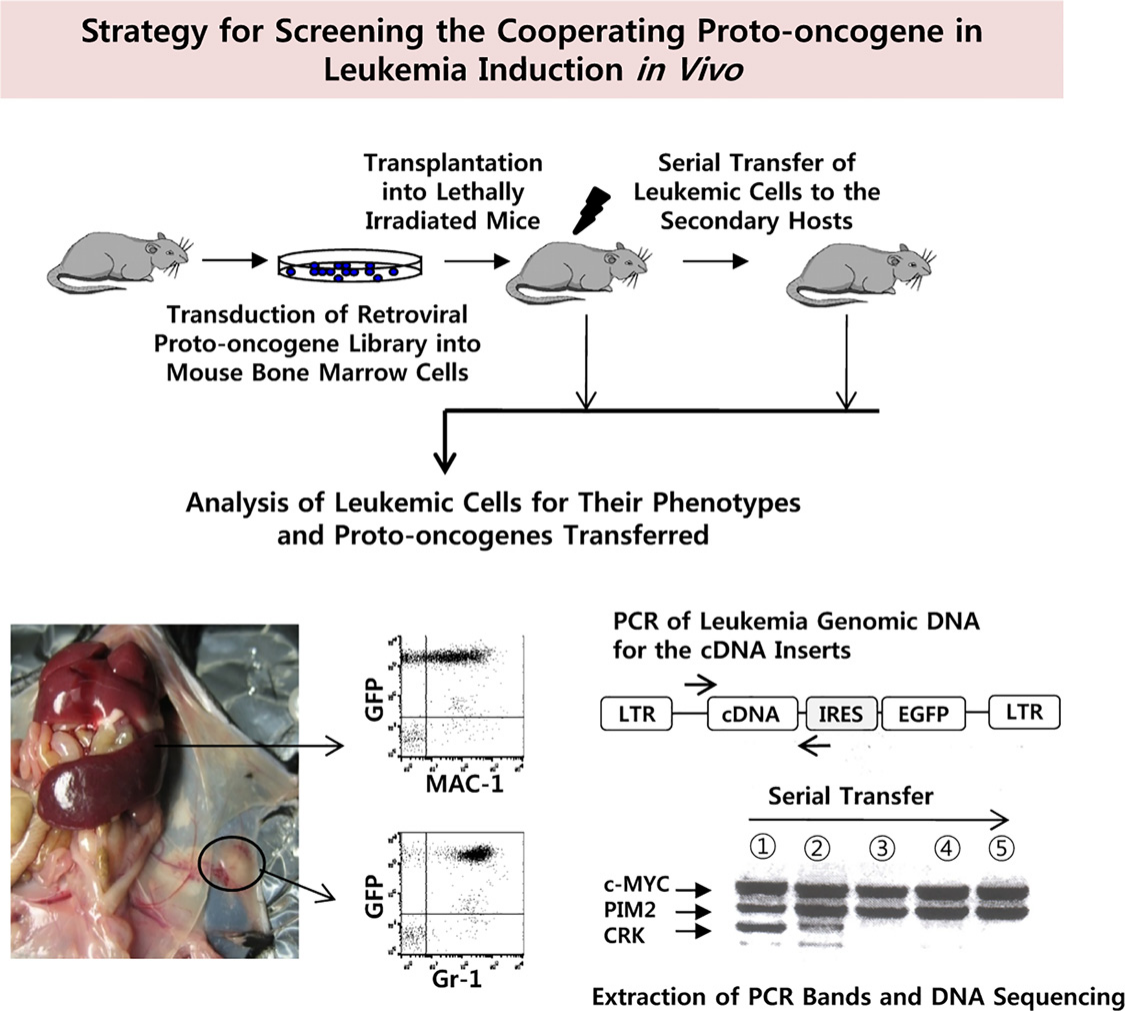
Screening Strategy for Proto-oncogene/homeobox Gene Combinations that are Leukemogenic *in vivo*. After transducing total bone marrow cells with a mixture of retroviral cDNA library, cells were transplanted into host mice. The mice showing characteristic leukemia symptoms were sacrificed, and leukemic cells were analyzed for surface phenotype. The cDNA inserts were analyzed by PCR amplification of the genomic DNA obtained from leukemic cells and subsequent nucleotide sequencing of the individual PCR product band excised from the agarose gels. In some experiments, the leukemic cells obtained from the primary leukemic mouse were transferred to the secondary host to enrich for the leukemic cells that are dominantly expanding *in vivo*.

Our strategy was to use a cDNA library of a defined set of proto-oncogenes and homeobox family genes to assay for leukemia-inducing genes or gene combinations. The cDNAs, integrated with the provirus, can easily be identified by PCR amplification of cDNA inserts from leukemic cells arising in transplanted animals, as shown in Fig 1. We reasoned that the leukemic cells will compete with each other and become oligo-clonal as the leukemic cells are serially transferred to the new hosts. As a result, the band pattern of amplified cDNA fragments become simpler as the serial transplantation continues although integration events could be assessed at any stage. The identity of the retroviral integrant can be elucidated by nucleotide sequencing of the PCR fragment excised from the agarose gel. We were able to establish permanent cell lines from each mouse and confirmed that the cell lines maintain the same cDNA inserts as the leukemic cells of the parental mice.

### Construction of a retroviral proto-oncogene cDNA library

**Fig 2:**
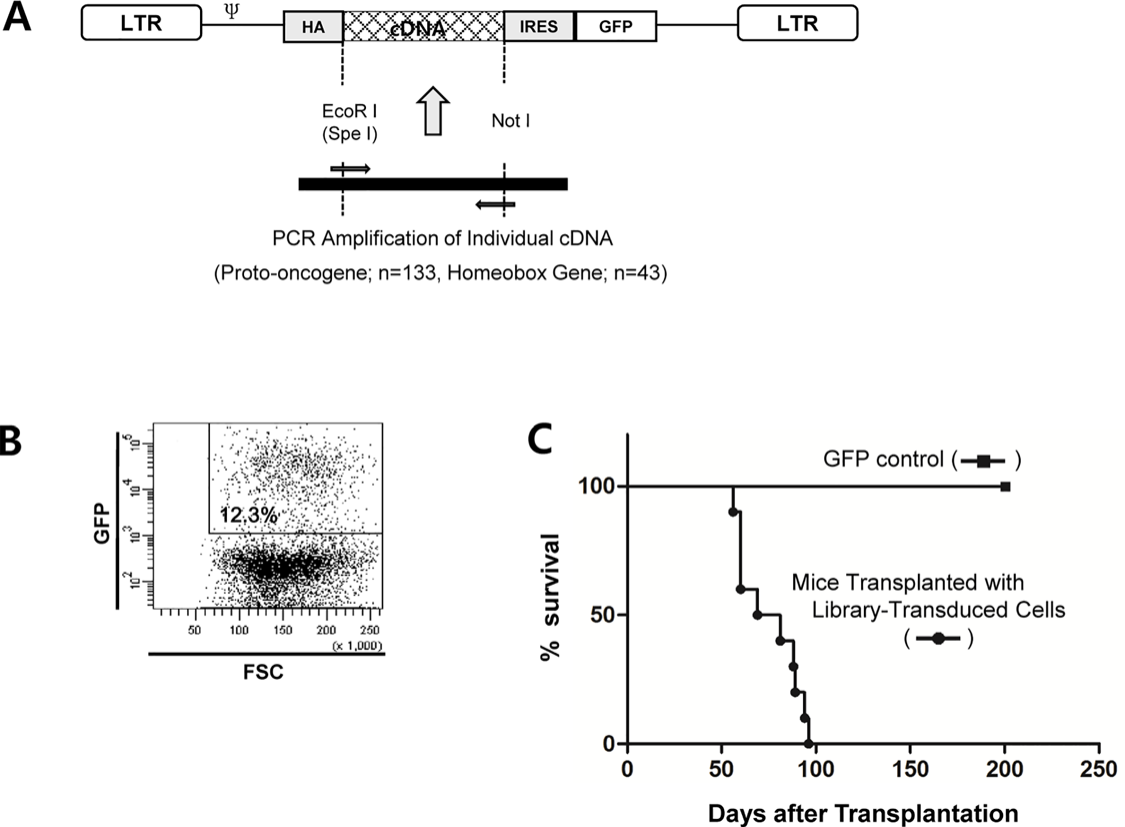
Construction of a Retroviral cDNA Library and Transduction/transplantation Study. **(A)** The structure of retroviral cDNA construct. The cDNAs of proto-oncogene and homeobox gene families were obtained from Mammalian Gene Collection (MGC). Individual cDNA was amplified by PCR with the corresponding primers synthesized for each cDNA to include an HA tag in the 5’ position. The 5’ and 3’ UTRs were removed by amplifying the coding sequences only. To produce the retroviral mixture, we mixed a constant amount (0.1 μg for each cDNA) of retroviral plasmid for every cDNA, and the cDNA mixture was transfected into the 293T cells along with ecotropic packaging plasmid pIK6.1MCV.ecopac.UTD (Ecopac: M. Finer Cell Genosys, Redwood City, CA). **(B)** We generally achieved 10 to 30% infection rate using the fresh mouse bone marrow cells as the target. Four μg/ml polybrene was included in the transfection medium along with the cytokine mixture described in Materials and Method. **(C)** Survival kinetics of mice transplanted with bone marrow cells transduced with the retroviral cDNA library. One million retrovirus-transduced cells were transplanted into syngenic γ-irradiated host mice through tail vain. Ten host mice were transplanted with the virus-transduced cells, and the mice showing the leukemic symptoms were sacrificed and the leukemic cells were analyzed in terms of surface phenotype and cDNA insert(s). The control mice (n = 10) were transplanted with bone marrow cells transduced with MSCV.IRES.GFP. No mice in the control group showed any sign of illness during the observation period (200 days).

Although the genes responsible for leukemia initiation can be screened with whole genome cDNA library, the increased complexity in cDNA library will decrease the possibility of discovering particular leukemogenic cDNA combinations. Therefore, the realistic alternative approach may be to use defined sets of gene families that are most likely contribute to leukemia initiation. We constructed a retroviral proto-oncogene cDNA library from the plasmid constructs obtained from the Mammalian Gene Collection (MGC) library (S1 Table). In total, 133 proto-oncogenes and 43 homeobox family gene cDNAs were individually cloned into MSCV retroviral vector in which GFP was co-expressed through an internal ribosome entry site (IRES). The HA-tag was inserted into the 5’end of the cDNAs, and all the 3’ and 5’ untranslated regions of the cDNAs were removed during the PCR amplification procedure (Fig 2A). To produce a retroviral cDNA library, a constant amount (0.1 μg) of individual retroviral construct plasmid was mixed first, and the resulting cDNA mixture was used to produce recombinant retrovirus using the Ecopac packaging system.

### Analysis of bone marrow transplants

The efficient transduction of retroviral cDNA library into fresh bone marrow cells requires certain degree of cell cycle progression and progenitor/stem cell enrichment. For this purpose, we conditioned mice with 5-FU, and the bone marrow cells were cultured in the presence of cytokine mixture. To minimize the cellular differentiation and unbalanced expansion of particular progenitor population of cells, we limited the *in vitro* culture period to 3 days during the retroviral infection process prior to *in vivo* transplantation. After transduction with the retroviral cDNA library, the initial transduction rate of bone marrow cells was determined by FACS analysis. As shown in Fig 2B, the GFP^+^ cells reached 12.3% at the time of primary bone marrow transplantation in a representative experiment. The bone marrow transduction and transplantation experiments were performed twice. In an initial experiment with retroviral cDNA library, all ten mice transplanted with cDNA library-transduced bone marrow cells developed leukemia and/or lymphoma within approximately 8 to 12 weeks (Fig 2C). When the host mice showed signs of leukemic disease such as hind limb paralysis, ruffled fur, hunched back and labored breathing, the mice were sacrificed and the phenotypes of the leukemic cells were analyzed. At the same time, the mixtures of spleen and BM cells from the sacrificed mice were serially transplanted into secondary host mice, and the leukemic cells were analyzed again after the mice showed signs of illness. The serial transfer was repeated two to five times depending on the mouse line. The leukemic cells were analyzed both in terms of surface phenotype and the identity of the cDNAs transferred

**Fig 3:**
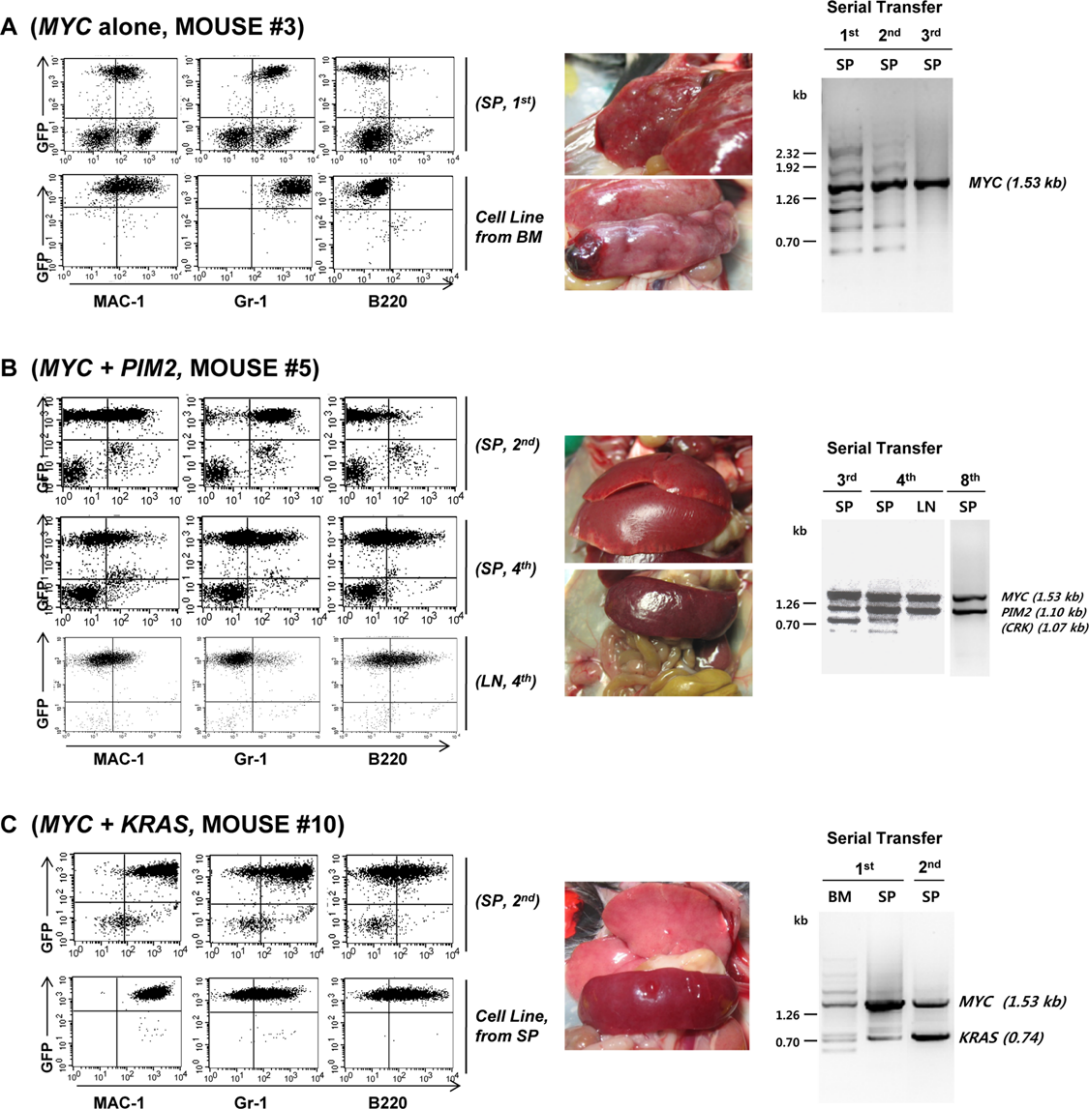
Characterization of the Leukemic Mice. **(A)** Phenotype of the leukemic cells of the host mice that have *MYC* as an only proto-oncogene insert transferred by retroviral cDNA library transduction. The phenotype of the leukemic cells obtained from Mouse #3 was shown as a representative result. The leukemic cells from Mouse #7 and Mouse #9 also have *MYC* cDNA insert only, and the results were shown in S1 Fig. The spleen cells from Mouse #3 were stained for MAC-1, Gr-1, and B220. The genomic DNA prepared from the spleen cells was used as a template to amplify cDNA insert(s), and the PCR product(s) was separated in agarose gel. The PCR product DNA bands were excised from the gel and the identity was determined by DNA sequencing. BM: bone marrow, SP: spleen, 1^st^: cells obtained from the 1^st^ host mice, 2^nd^: cells obtained from the secondary host mice after serial bone marrow transfer. **(B)** A representative phenotype of the mice harboring *MYC* and *PIM2* proto-oncogene in their leukemic cells (Mouse #5). The phenotype of the leukemic cells from Mouse #4 and Mouse #8 are shown in S2 Fig 3^rd^, 4^th^, 8^th^: Cells obtained from the host mice after 3^rd^, 4^th^, and 8^th^ serial transfer. LN: Lymph Node. **(C)** A representative phenotype of the mice harboring *MYC* and one additional cDNA insert of which the role in leukemogenesis is uncertain. The result from Mouse #10 harboring *KRAS* and *MYC* cDNA inserts is shown. The phenotypes of the additional three mice harboring *PDGFB*, *RAB7B*, or *CRX* in addition to *MYC* are shown in S3 Fig.

Based on the cDNA transgenes retained by the leukemic cells, we classified the mice into three groups. The first group included three mice (mice #3, #7, #9) in which the only cDNA identified as a transgene in their leukemic cells was *MYC*. The data on mouse #7 and #9 were presented in Supporting Information (S1 Fig). As shown in Fig 3A and S1 Fig, the mice in this group all showed enlarged liver and spleen with conspicuous white nodules full of leukemic cells. The leukemic cells of these mice all showed a myeloid phenotype, even after repeated serial transfer into the lethally irradiated host mice. Although the *MYC* transgene we used in this study was of human origin, these data confirm the conclusion of a previous study[9] in which ectopic expression of *c-MYC* (murine origin) in mouse bone marrow cells induces acute myeloid leukemia after transplantation of transduced cells into lethally irradiated mice. In addition, gradual dominance of *MYC* during serial transplantation was evident in mice #3 and #7, in which the band pattern of amplified cDNA fragment became simplified as the serial transplantation was continued. For all these mice, permanent cell lines were easily established even in the absence of exogenous cytokines in the culture medium.

The second group included mice with leukemia cells harboring two proto-oncogenes known to act in synergistic combination in cancer maintenance and induction, *MYC* and *PIM2*[10–13]. As shown in Fig 3B, mouse #5 with *MYC* and *PIM2* proto-oncogenes showed enlarged spleen and extensive lymphoma. The leukemic cells initially showed myeloid phenotype of high level of MAC-1 and Gr-1 expression with basal level of B220. After serial transplantation, the cells acquired phenotype of MAC-1^low^, Gr-1^low^, B220^low^, and this obscure phenotype is more obvious in the cell line established from the bone marrow of the first generation host mouse. On the other hand, mouse #4 with *MYC* and *PIM2* proto-oncogenes developed extensive lymphoma in various anatomical locations, and the cells clearly showed the phenotype of pro-B cell lymphomas as judged by CD43 and membrane IgM staining (S2A Fig). Another *PIM2*/*MYC* mouse (mouse #8, S2B Fig) showed myeloid leukemia phenotype as judged by FACS analysis, and we were able to establish an *in vitro* cell line of myeloid phenotype (Gr-1^+^, Mac-1^+^, B220^-^). These results may indicate that *MYC* and *PIM2* can induce both AML and B-lineage lymphoma, and the outcome may depend on the initial target of viral infection.

The third group of mice harbors *M*YC and one additional proto-oncogene for which the role cannot be clearly defined in the present system because of the extremely potent leukemogenic potential of *MYC* itself. Fig 3C shows mice with leukemic cells harboring *MYC* and *KRAS*. The proto-oncogenes *MYC* and *KRAS* are known to be the most prominent combination of synergistic oncogenic potential in various solid tumors [14,15]. However, the synergistic nature of *MYC* and *KRAS* in leukemia induction has only been indicated in an indirect manner through the frequent occurrence of *KRAS* mutation and elevated MYC expression [16] in leukemic cells of AML patients. As shown in Fig 3C, the leukemic cells from the *MYC*/*KRAS* mouse (mouse #10) showed a somewhat obscure phenotype simultaneously expressing Mac-1, Gr-1 and B220. The established cultured cell line from this mouse also showed a similar phenotype of Mac-1^+^/Gr-1^+^/B220^+^. The additional proto-oncogenes identified in this group included *PDGFB* (S3A Fig, Mouse #1), *RAB7B* (S3B Fig, Mouse #2), and *CRX* (S3C Fig, Mouse #6), and the leukemic cells from these mice all showed a myeloid phenotype. Whether these genes are truly synergistic with *MYC* in leukemia induction remains to be determined in future studies.

### Leukemia induction by *MYC*-deleted library showed *MEIS1* and *HOX* family genes as major leukemogenic gene combination

**Fig 4:**
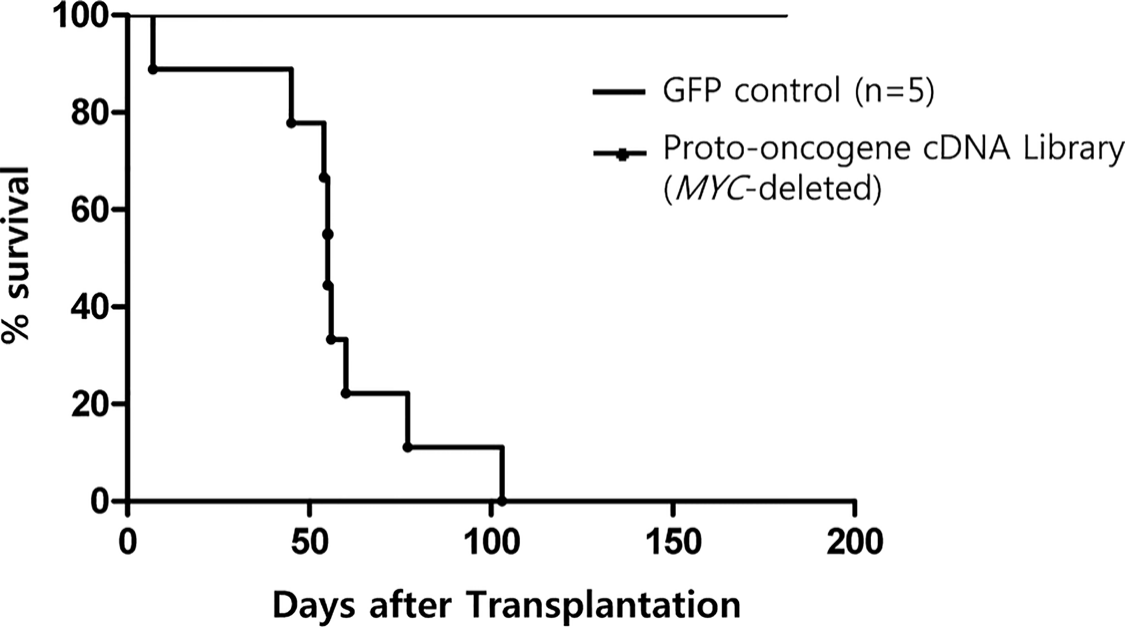
Survival Kinetics of the Mice Transplanted with Bone Marrow Cells Transduced with the *MYC*-deleted Retroviral cDNA Library. The mice (n = 9) transplanted with the *MYC*-deleted library all succumbed to leukemia within 8 to 15 weeks after transplantation of bone marrow cells transduced with retroviral cDNA library in which six *MYC*-related genes were excluded from the original library. The mice showing signs of leukemic disease were euthanized and the leukemic cells were analyzed as described in Materials and Method. Again, the control group (n = 5) did not show any sign of illness during the observation period. Among the 9 host mice, one mouse was found to be dead on the previous day (Day 7) and further analysis was not performed.

**Fig 5:**
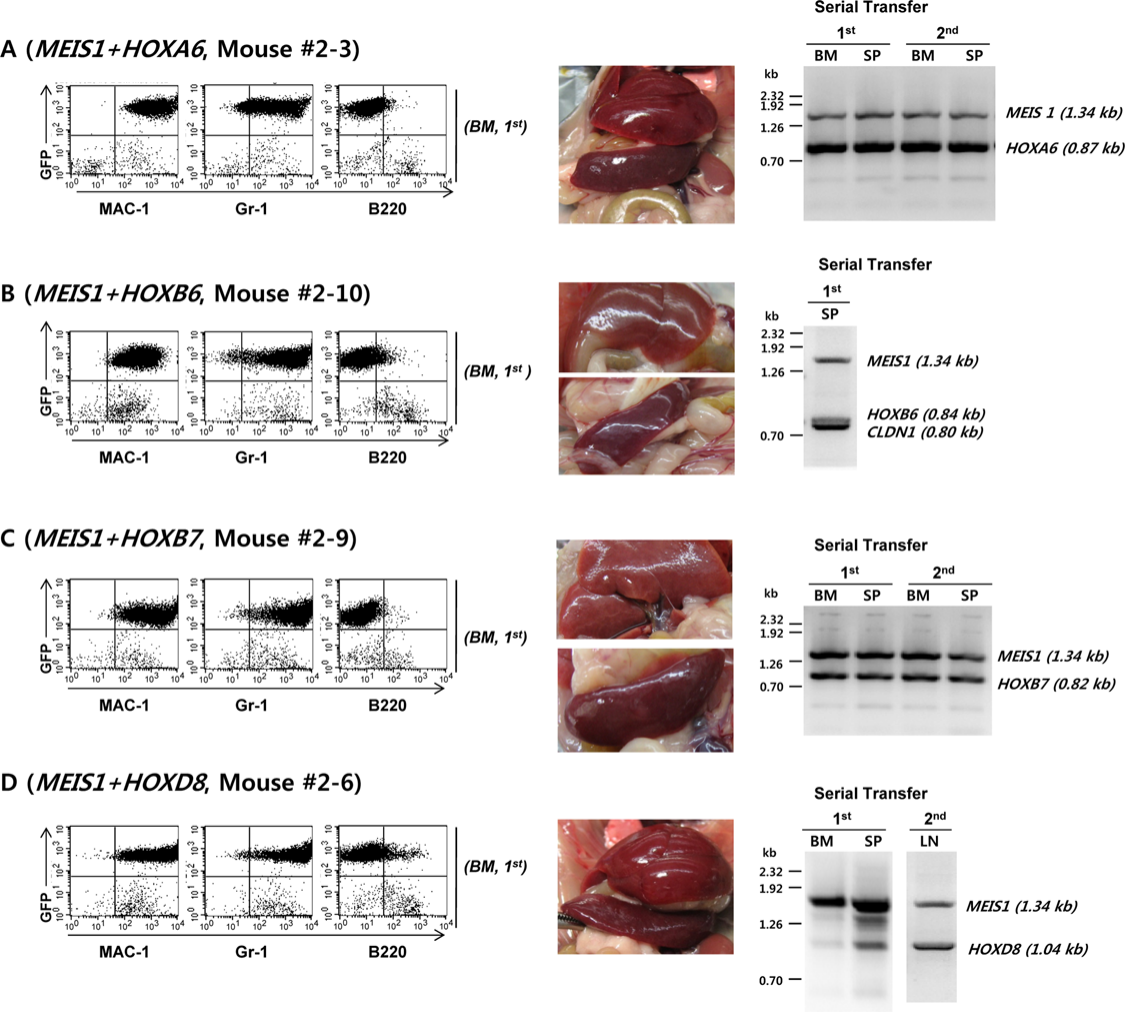
The Mice Transplanted with Bone Marrow Cells Transduced with *MYC*-deleted Retroviral cDNA Library Developed Acute Myeloid Leukemia Harboring *MEIS1* and One of Four *HOX* cDNA Inserts. The leukemic cells from the 8 mice that have developed leukemia were analyzed in terms of their surface phenotype and cDNA inserts. The bone marrow cells were transduced with the retroviral cDNA library supernatant on Day 1 and again on Day 2. One million retrovirus-transduced bone marrow cells were transplanted into 9 irradiated host mice. One mouse died at Day 7 with unknown cause and was excluded from the analysis. **A**. Mouse 2–3 (*MEIS1* + *HOXA6*), **B**. Mouse 2–10 (*MEIS1* + *HOXB6*), **C**. Mouse 2–9 (*MEIS1* + *HOXB7*). **D**. Mouse 2–6 (*MEIS1* + *HOXD8*).

We tried to confirm cooperation between MYC and the candidate cooperative genes such as *KRAS*, *PDGFB*, *RAB7B*, *CRX* that have been described in Fig 3. However, the strong leukemogenic potential of *MYC* itself did not allow proper analysis of cooperative potential of additional genes. Therefore, we decided to remove *MYC*-related genes from the cDNA library in an effort to reveal additional leukemogenic gene combinations (See S2 Table for the list of deleted *MYC*-related proto-oncogenes). As shown in Fig 4, the transduction of *MYC*-deleted library into mouse bone marrow cells and subsequent transplantation induced acute myeloid leukemia in all the host mice within 6 to 15 weeks. As in the previous experiment, the control mice that received the GFP transgene alone remained healthy throughout the observation period (6 months). Most of the treated mice showed signs of leukemic disease within 60 days, and the phenotype of the leukemic cells was uniformly myeloid as judged by the surface staining and FACS analysis (Fig 5).

The importance of *CLDN1* (in Mouse 2–10) cDNA insert in AML induction is not clear. All the mice showed AML-type leukemia and myeloid sarcoma. The phenotypes of the leukemic cells from the four additional mice harboring *MEIS1* and *HOXA6*, *HOXB7*, or *HOXD8* are shown in Supporting Information (S4 Fig). Based on the cDNA inserts of leukemic cells, we divided the mice into four groups. The first group of mice had *MEIS1* and *HOXA6* as their main cDNA inserts (Fig 5A, Mouse #2–3). Although *PIM1*, *CLDN1* (S4A Fig, Mouse #2–5), and *PIM3* (S4B Fig, Mouse #2–7) proto-oncogenes were identified in our screen, their role in leukemia induction is not clear given the well-known co-operative nature of *MEIS1* and anterior Hox genes such as *HoxA7*, *8*, *9*[17–20]. Although the stimulatory effect of *HOXA6* on bone marrow cell proliferation has been previously documented [21,22], this is the first report of AML induction in mice by *HOXA6* in co-operation with *MEIS1*. The second group includes the mouse with *MEIS1* and *HOXB6* (Fig 5B, Mouse #2–10). Since it is well known that *MEIS1* and *HOXB6* synergistically cooperate in leukemia induction [23–26], the detection of *MEIS1/HOXB6* combination among the leukemic mice further validates the usefulness of the current model system. Although the *CLDN1* band is present in addition to *HOXB6*, the role of *CLDN1* in leukemia initiation is not clear and needs further investigation. The third group of mice harbors *MEIS1* and *HOXB7* (Fig 5C and S4C Fig). The *MEIS1* and *HOXB7* combination has never been reported to have leukemogenic potential before. The fourth group possessed *MEIS1* and *HOXD8* as the major cDNA inserts in their leukemic cells (Fig 5D and S4D Fig). *HOXD8* has never been reported to be involved in cancer initiation before, so this is the first report of cooperation between *HOXD8* and *MEIS1* in AML induction. Again, there were additional cDNA inserts, *RAB39B*, in addition to *MEIS1* and *HOXD8* (S4B Fig), and the importance of its role is not clear at this moment.

### Confirmation of the synergistic effect of *MEIS1*-*HOXB7* and *MEIS1*-*HOXD8* in leukemia induction

**Fig 6:**
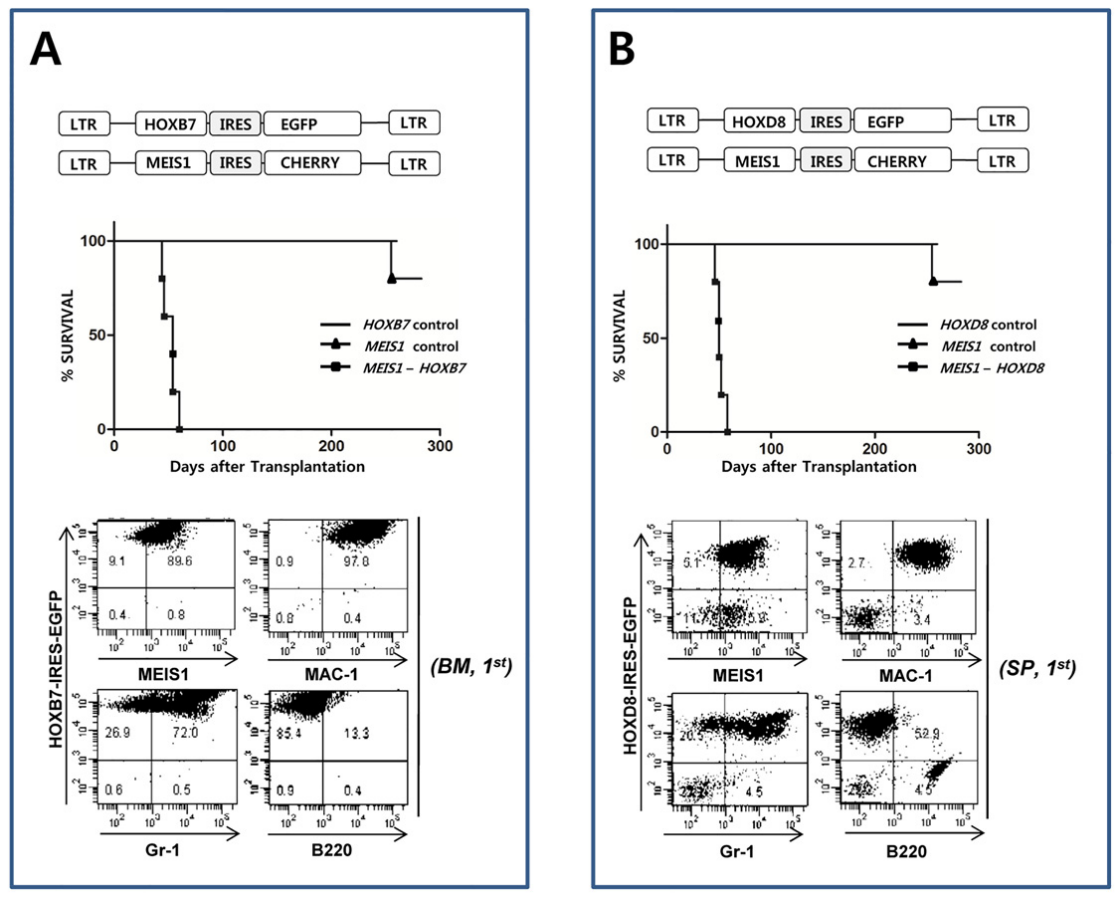
The Synergistic Effect of *MEIS1* with *HOXB7* or with *HOXD8* in Leukemia Induction. To confirm the synergistic effect of *MEIS1* with *HOXB7* or with *HOXD8*, *MEIS1* was inserted into the IRES-Cherrypicker MSCV retroviral vector (Clontech) to visualize the *MEIS1*-expressing cells by surface staining of the Cherrypicker marker protein. As in cDNA library, *HOX* gene was expressed from MSCV-IRES-GFP retroviral vector. The bone marrow cell transduction and transplantation was done as described in Materials and Method except that mixtures of retroviral supernatants of *MEIS1/HOXD8* or *MEIS1/HOXB7* were used for infection instead of cDNA library supernatant. **A**. Co-operation of *MEIS1* and *HOXB7* in leukemia induction. Co-transduction of bone marrow cells and subsequent transplantation of the transduced cells into host mice induced leukemia in the recipients within 60 days (n = 5). Most of the leukemic cells co-express the *MEIS1* and *HOX* genes, and the cells are of myeloid phenotype. *MEIS1* or *HOXB7*alone did not induce leukemia for more than six months (n = 5). **B**. Co-operation of MEIS1 and HOXD8 in leukemia induction. Co-transduction of bone marrow cells and subsequent transplantation of the transduced cells into host mice induced leukemia in the recipients within 60 days (n = 5). As shown in the figure, *MEIS1* or *HOXD8* alone did not induce leukemia for more than six months (n = 5).

The results from Fig 4 and Fig 5 showed two genes, HOXB7 and HOXD8 as candidate gens that are cooperating with MEIS1 in leukemia induction. To verify the screening results, the synergistic effect of *MEIS1* with *HOXB7* or *HOXD8* in leukemia induction was tested in a bone marrow transduction and transplantation experiment. As shown in Fig 6A (*HOXB7*) and Fig 6B (*HOXD8*), the co-transduction of *MEIS1* with *HOXB7* or *HOXD8* exerted a strong synergistic effect in leukemia induction. In both cases, the leukemic cells were of myeloid phenotype and all the mice died of leukemia within 60 days. Transduction of *MEIS1*, *HOXB7 or HOXD8* alone did not induce leukemic disease during the observation period of 6 months. One mouse died in *MEIS* alone group showed no sign of leukemic disease such as splenomegaly or enlarged lymph node by visual inspection during autopsy. These results strongly validate the results obtained from the cDNA library transduction experiments described in this report. As previously reported, *MEIS1* alone did not induce leukemia [27,28].

## Discussion

Until recently, the identification of genes responsible for leukemia induction has relied on the cloning of chromosomal breakpoints and the analysis of mutated or over-expressed genes in leukemic cells. Although scores of fusion genes and over-expressed or mutated genes important in leukemia initiation have been elucidated from these studies, a systematic screening system for detection of leukemogenic genes or gene combinations in an experimental animal model will be invaluable in elucidating novel leukemogenic genes or gene combinations to complement the studies on clinical samples. One such strategy [29,30] exploits the radiation-induced mutagenesis and unique event in the retrovirus life cycle: random integration of proviral DNA into chromosomal DNA. In this strategy, bone marrow cells were first irradiated with γ-radiation and then transduced with retroviral vector containing no functional gene. In very rare cases, the proviral sequences integrate into the 5’ position of presumably leukemogenic proto-oncogenes, resulting in overexpression of corresponding proto-oncogenes in downstream position, thus initiating leukemia development. The *PIM* oncogenes were discovered using this strategy [4,31]. However, the strategy suffers from several shortcomings. First, since the strategy relies on the rare event of random integration of proviral DNA and subsequent over-expression of a downstream proto-oncogene influenced by the retroviral LTR enhancer, the strategy inherently needs a large sample size. Second, since the chance of two rare events occurring in a single cell is very low, the strategy can screen only for individual genes, not for gene combinations. In addition, the secondary mutation events inflicted by γ-irradiation itself may never be known.

In the present study, we developed an animal model system in which mouse bone marrow cells were transduced with a retroviral cDNA library composed of a pre-determined gene pool. The transduced cells were then transplanted into lethally irradiated syngenic recipient mice. The mice that developed leukemia were sacrificed, and the identity of the genes transferred to leukemic cells were characterized by PCR amplification and subsequent sequencing of retroviral cDNAs inserted into genomic DNAs of leukemic cells.

To test the usefulness of the model system, we used a proto-oncogene and homeobox gene retroviral cDNA library. The current model described in this study allows leukemia development only by the expression of cDNAs delivered by retroviral transduction. The first screen of the proto-oncogene/homeobox gene pool produced ten mice with leukemic cells with *MYC* as a common cDNA insert. Among the ten mice, three (Mouse #3, #7, #9) had leukemic cells with *MYC* as the only cDNA insert in their genome. Although there has been controversy about the ability of *MYC* to induce leukemia on its own [9],[32], our result clearly shows that *MYC*, when over-expressed, can initiate leukemia without any additional genetic change.

The second group of three mice (Mouse #4, #5, #8) was shown to harbor *PIM2* as a secondary proto-oncogene cDNA in addition to *MYC*. The *PIM2* and *MYC* combination of proto-oncogenes is known to have a synergistic effect on prostate carcinoma [33] and B-lymphoma development [11]. The appearance of *PIM2* and *MYC* together in three different mice strongly suggests that the two proto-oncogenes synergistically stimulate leukemia development. The Mouse #5 (Fig 3B) initially developed myeloid type of leukemia and lymphoma. After serial transfer of the bone marrow cells into the syngenic host mice, the leukemic cells gradually acquired B220 surface marker. In particular, the loss of myeloid marker expression was apparent among the lymphoma cells harvested from the enlarged lymph node nodules. The leukemic cells from another mouse of *PIM2/MYC* group (S2A Fig Mouse #4) initially showed obscure phenotype of Gr-1^+^, Mac-1^+^, and B220^+^. The cell lines established from the spleen of this mouse showed pro B cell phenotype according to the surface IgM, CD117, and CD43 staining[34,35]. On the other hand, the leukemic cells from the third mouse of this group, mouse 8, showed myeloid phenotype expressing Mac-1 and Gr-1 only. These results show that the *PIM2* and *MYC* combination might have potential to drive leukemic transformation of either myeloid or lymphoid progenitor cells.

The third group includes four mice with leukemic cells harboring additional cDNA inserts in addition to *MYC*. The leukemic cells from the mice in Fig 3C (Mouse #10) has *KRAS* in addition to *MYC*, and show obscure phenotype of Mac-1^+^, Gr-1^+^, and B220^+^. The similar phenotype was observed in the cell line established from the spleen of the same mice. Since the strong cooperation of *MYC* and *RAS* has been reported before in various solid tumors [33], the *MYC/KRAS* combination observed in Mouse #10 most likely reflects the outcome of genuine cooperation between the two genes in leukemia induction. Although we performed transduction/transplantation experiments to clarify this point, the strong leukemogenic potential of *MYC* alone did not allow us to make definite conclusion. The cooperation between *MYC* and the additional three genes shown in S3 Fig, *PDGFB* (S3A Fig, Mouse #1), *RAB7B* (S3B Fig, Mouse #2), *CRX* (S3C Fig, Mouse #6), cannot be adequately analyzed due to the same reason.

Since the results in the first part of experiment show that the *MYC* proto-oncogene is extremely potent in initiating hematopoietic cancer, we deleted *MYC*-related genes from the library to further elucidate leukemogenic proto-oncogene combinations that might have been obscured in the presence of *MYC*. The eight mice transplanted with bone marrow cells transduced with *MYC*-deleted proto-oncogene retroviral cDNA library all succumbed to myeloid leukemia within eight weeks of transplantation (Fig 4). The analysis of cDNA inserts in the leukemic cells showed that all eight leukemic mice contained *MEIS1* and at least one HOX class 1 family gene. The cooperation between *HOXB6* and *MEIS1* in leukemogenesis has been reported before [23,26],[36].

*HOXB7* and *HOXD8* are new partners of *MEIS1* that have never been reported to have leukemia-inducing potential prior to this study. We further confirmed that *MEIS1* and *HOXB7* or *MEIS1* and *HOXD8* combinations are genuinely synergistic in leukemia induction (Fig 6). On the other hand, either gene alone did not induce leukemia in the transduction/transplantation study during the observation period of 180 days.

There are several technical issues that must be resolved to establish the present model as an efficient screening system for leukemogenic (or tumorigenic) gene combinations. First, the PCR identification of the cDNA inserts in leukemic cells must be highly accurate and efficient. To prove this point, we submitted (S3 Table) graphical sequencing profile of the every cDNA band from Mouse #1 to Mouse #5 in the first set of experiment in which leukemia was initiated with retroviral cDNA library (including *MYC*). As shown in the table, all the major PCR bands were clearly readable except several faint minor bands in which multiple bands may coexist. Second, the PCR amplification products from cDNA library as well as from the virus-transduced cells must evenly represent members of the library. To prove this point, we carried out NGS (New Generation Sequencing) analysis for the PCR products amplified from the plasmid mixture of the cDNA library and genomic DNA from the bone marrow cells transduced with the retroviral cDNA library. As shown in S4 Table, we found the representation of individual cDNA in library is not as even as we expected. Furthermore, we were not able to detect PCR products corresponding to 12 cDNA members. This result may have been caused by the variation in plasmid amount during library construction or difficulty in amplifying certain cDNA sequences compared to others. However, we still think that the screening system we introduced is highly valuable with the more refined cDNA library of expanded gene sets from the commercial source in which the representation is more even and the expression is verified for every library member. Third, the system must avoid insertional mutation caused by too high MOI that may complicate the interpretation of the data. For this, we used MOI of 1 in all the experiment we performed. As shown in S5 Fig, the provirus integration events were limited. In addition, the current system is looking into the acute leukemia in which leukemia induction is complete in 8 to 10 weeks in most cases. Since the insertional mutation caused by provirus become apparent only after considerable incubation period of more than 6 months it is not likely that insertional mutation may affect experimental outcome. Fourth, the members of the library must express their cDNA in target cells. The efficient expression of each insert of the cDNA library is absolutely required for proper screening system. We did not check every ember of the library for their expression of the cDNA inserts. We did show protein expression of MYC, PIM2, MEIS1, HOXB7, and HOXD8 in leukemic mice (S6 Fig). However, more refined cDNA library in which proper expression of the every member of the library is evaluated and documented will be very useful in more extensive screening process in future.

In this study, we reported an animal model system in which leukemogenic gene combinations can be efficiently identified in a retroviral cDNA transduction and *in vivo* transplantation system. Although we tested the usefulness of the model system using a proto-oncogene/homeobox gene family retroviral cDNA library, the system can be applied to various other gene families that have been known to be important in the regulation of cell cycle progression, cell survival, and differentiation. The model system we described in this study can be used to characterize new combination of genes in cancer initiation of various tissue origins including hematopoietic stem cells and intestinal epithelial stem cells

## Supporting Information

S1 Fig Phenotype of the leukemic cells of the host mice that have MYC as an only proto-oncogene insert transferred by retroviral cDNA library transduction. (A) Mouse #7. (B) Mouse #9. BM: bone marrow, SP: spleen, 1^st^: cells obtained from the 1^st^ host mice, 2^nd^: cells obtained from the secondary host mice after serial bone marrow transfer.
(TIF)

S2 Fig The phenotype of the leukemic cells from Mouse 4 and Mouse 8. 1^st^, 3^rd^, 8^th^: Cells obtained from the host mice after 1^st^, 3^rd^, and 8^th^ serial transfer. LN: Lymph Node. Cell lines were established from the spleens of the leukemic cells.
(TIF)

S3 Fig Phenotype of the leukemic cells from the mice harboring *MYC* and one additional cDNA insert of which the role in leukemogenesis is uncertain. Refer to Fig 3C for detailed explanation.
(TIF)

S4 Fig Phenotype of the leukemic cells from the mice that have developed leukemia after transplantation of bone marrow cells transduced with *MYC*-deleted retroviral cDNA library. Refer to Fig 5 for detailed information. **A, B.** Mice harboring *MEIS1* and *HOXA6* (Mice #2–5 and #2–7).**.** Mouse harboring *MEIS1* and *HOXB7* (Mouse #2–4). **D.** Mouse harboring *MEIS1* and *HOXD8* (Mouse #2–8).
(TIF)

S5 Fig Southern blot analysis to reveal provirus integration events in genomic DNAs of the leukemic cells. (TIF)

S6 Fig Western blot analysis of the proto-oncogene expression in leukemic cells. (TIF)

S1 Table List of cDNAs included in Retroviral proto-oncogene and homeobox gene cDNA library. (DOCX)

S2 Table List of deleted MYC-related proto-oncogenes. (DOCX)

S3 Table Genomic DNA-PCR Sequencing profile. (DOCX)

S4 Table Result of Next Generation Sequencing (NGS). (DOCX)
