# Supplementary figures and images for: Characterization of Leukemia-Inducing Genes Using a Proto-Oncogene/Homeobox Gene Retroviral Human cDNA Library in a Mouse In Vivo Model
Source: PLoS One. 2015 Nov 25;10(11):e0143240. doi: 10.1371/journal.pone.0143240 (PMC4659616; doi:10.1371/journal.pone.0143240)

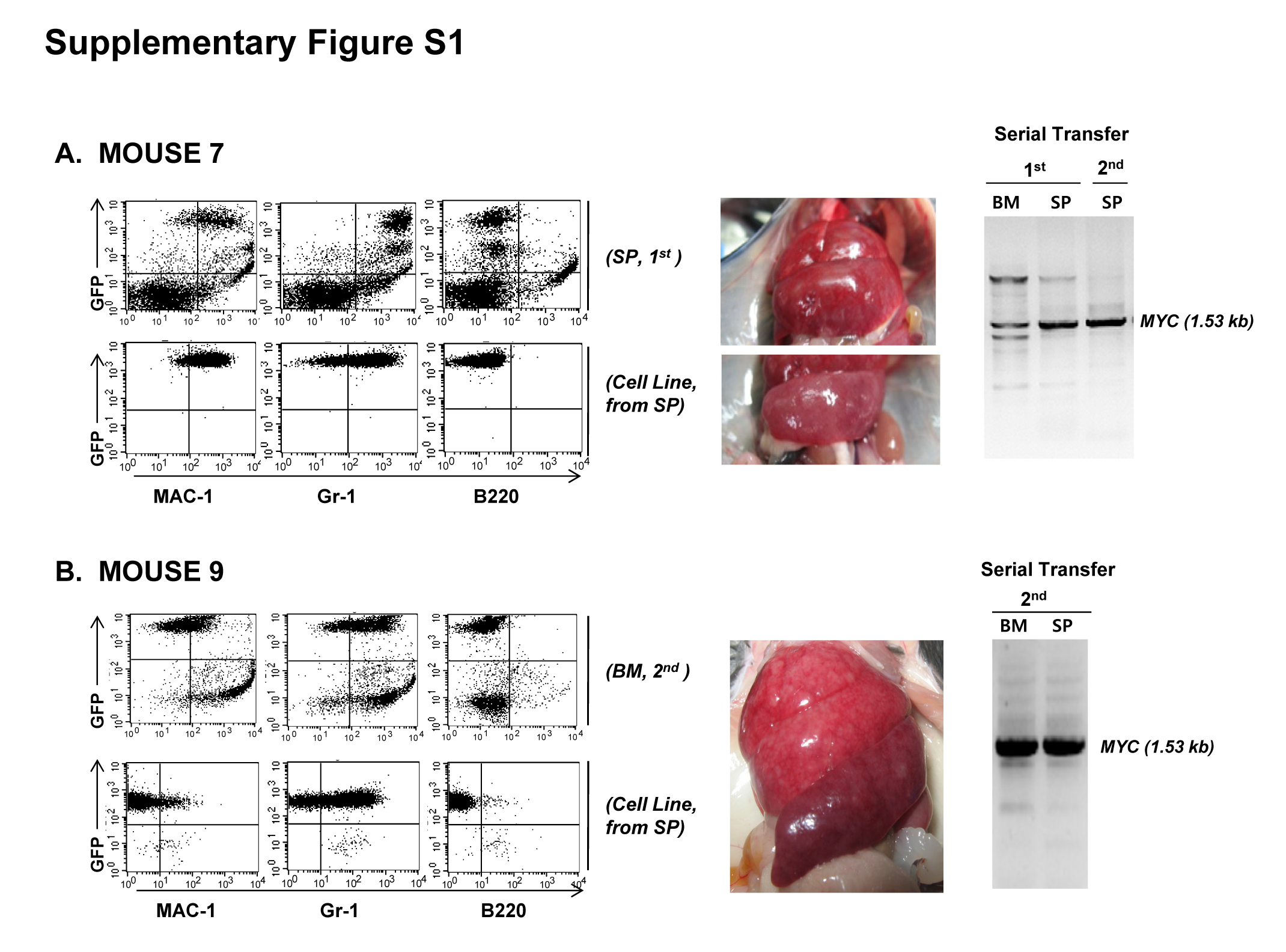

Supplement: S1 Fig — (A) Mouse #7. (B) Mouse #9. BM: bone marrow, SP: spleen, 1st: cells obtained from the 1st host mice, 2nd: cells obtained from the secondary host mice after serial bone marrow transfer. (TIF) [file pone.0143240.s001.tif]

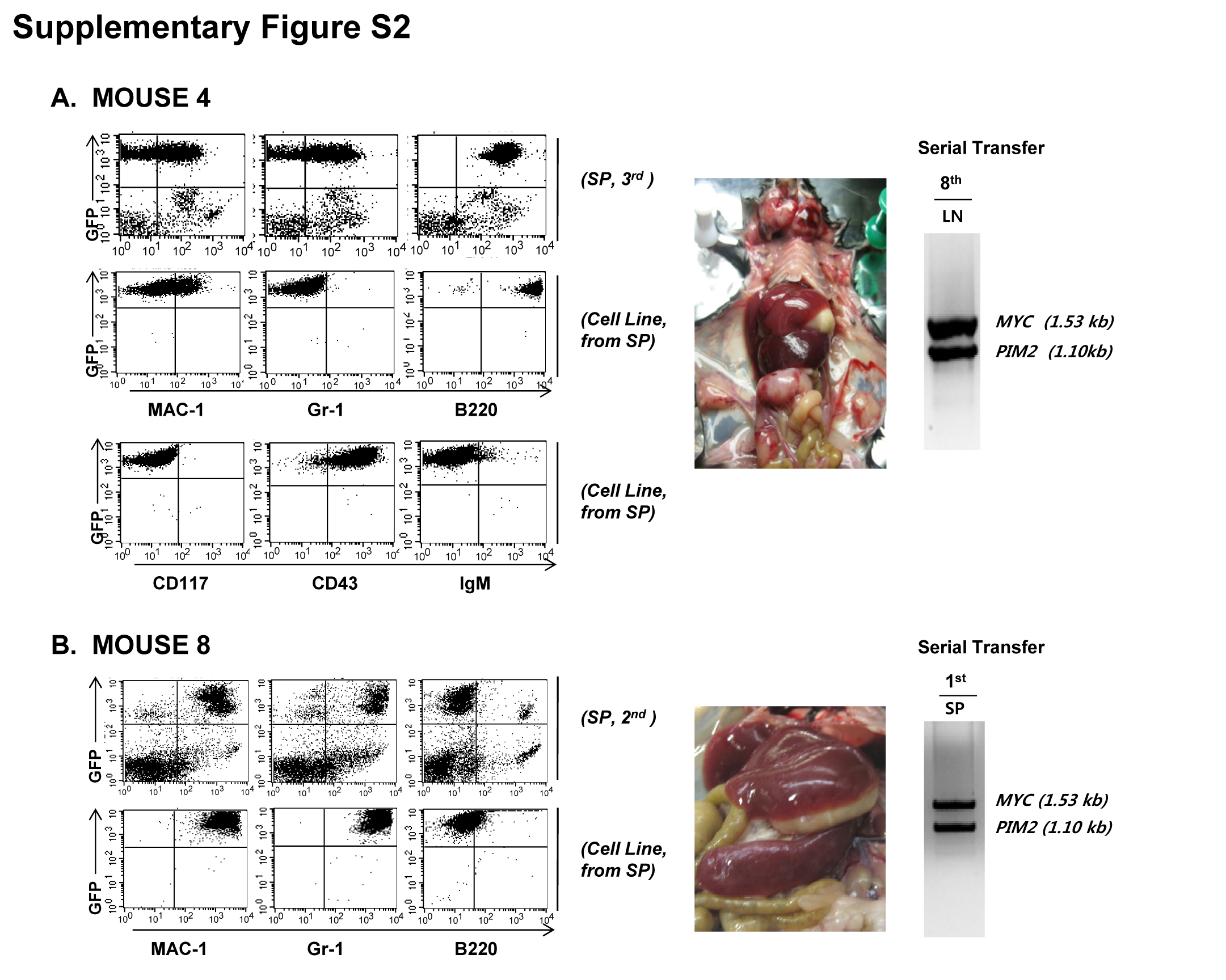

Supplement: S2 Fig — 1st, 3rd, 8th: Cells obtained from the host mice after 1st, 3rd, and 8th serial transfer. LN: Lymph Node. Cell lines were established from the spleens of the leukemic cells. (TIF) [file pone.0143240.s002.tif]

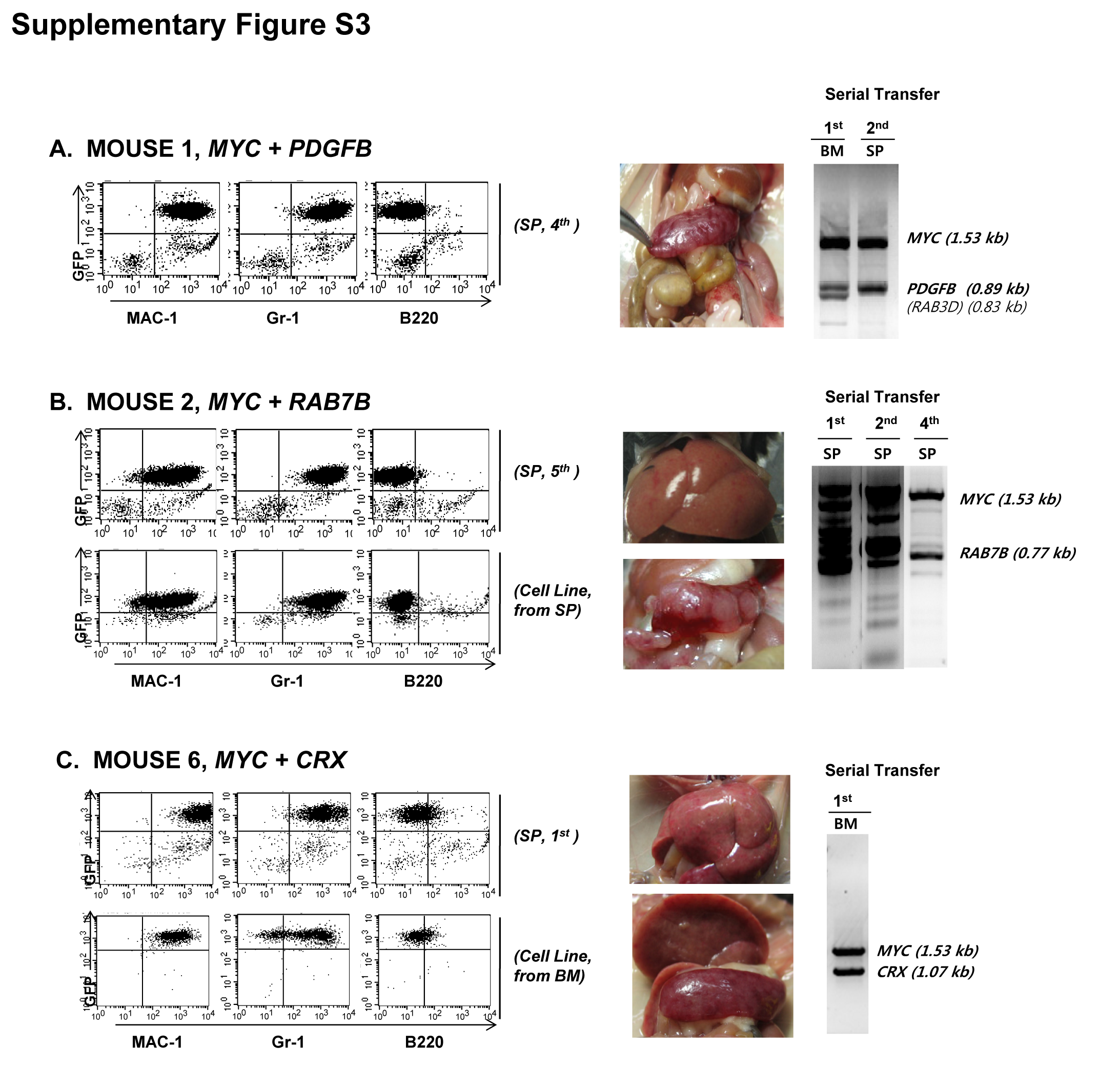

Supplement: S3 Fig — Refer to Fig 3C for detailed explanation. (TIF) [file pone.0143240.s003.tif]

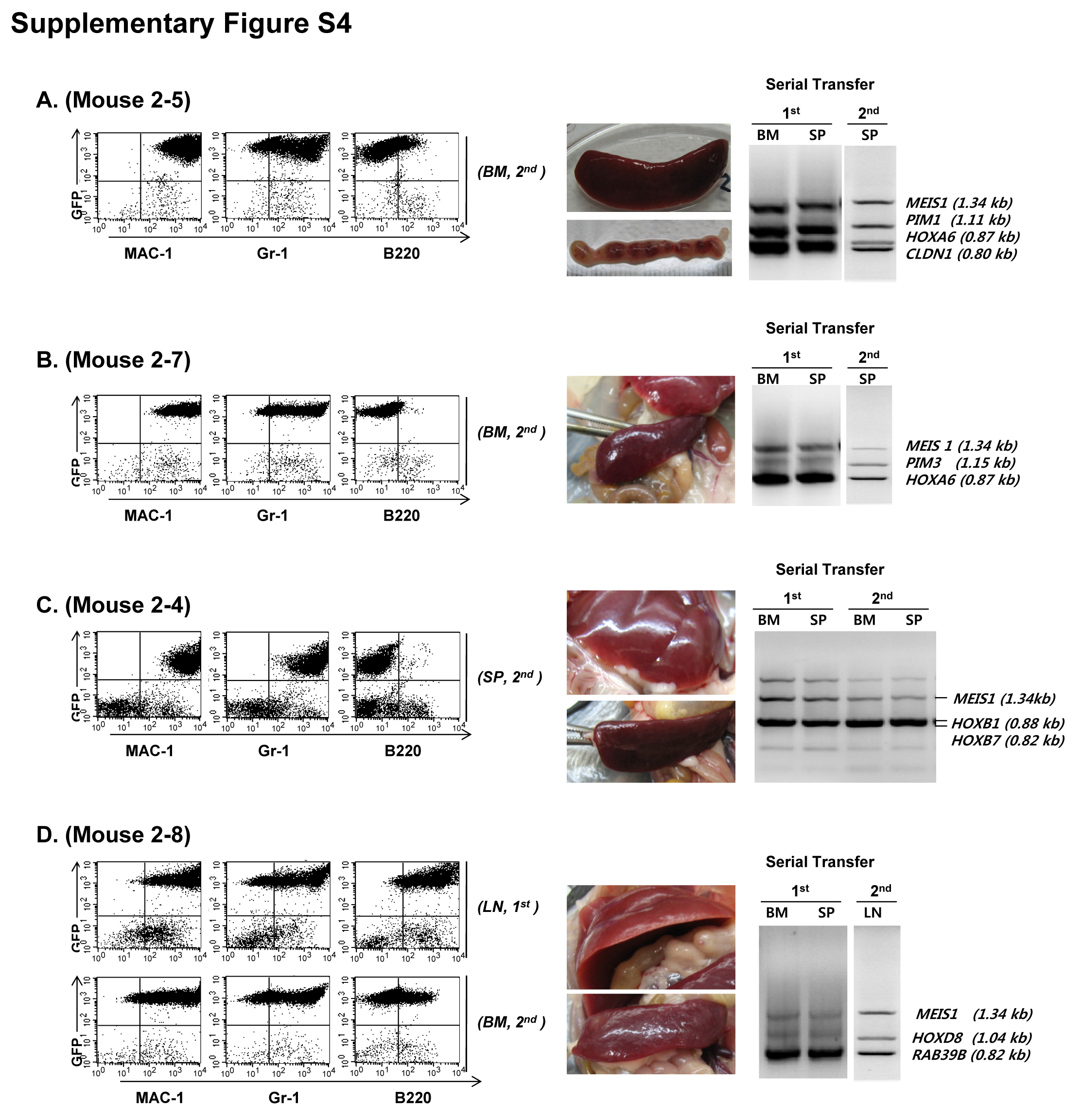

Supplement: S4 Fig — Refer to Fig 5 for detailed information. A, B. Mice harboring MEIS1 and HOXA6 (Mice #2–5 and #2–7).. Mouse harboring MEIS1 and HOXB7 (Mouse #2–4). D. Mouse harboring MEIS1 and HOXD8 (Mouse #2–8). (TIF) [file pone.0143240.s004.tif]

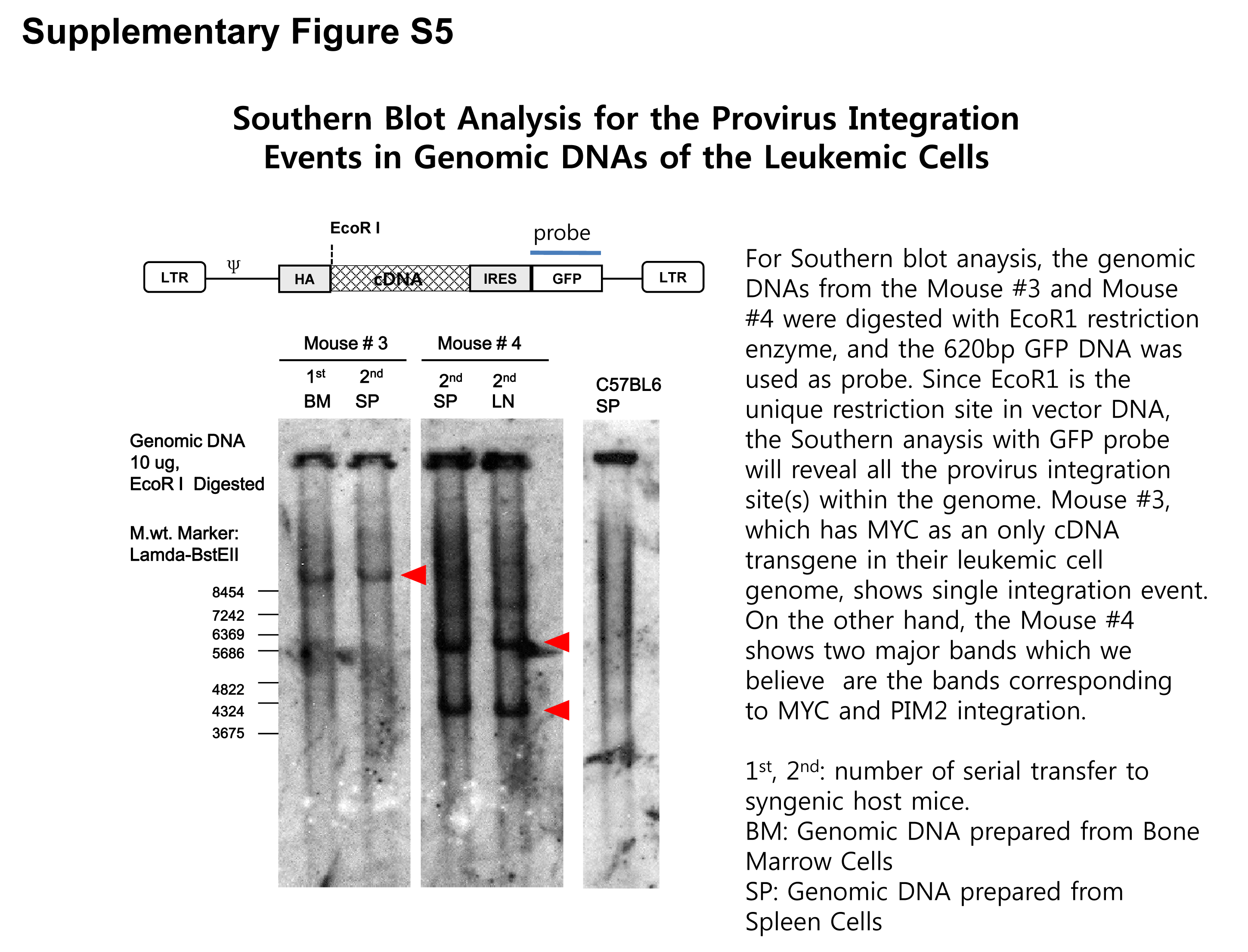

Supplement: S5 Fig — (TIF) [file pone.0143240.s005.tif]

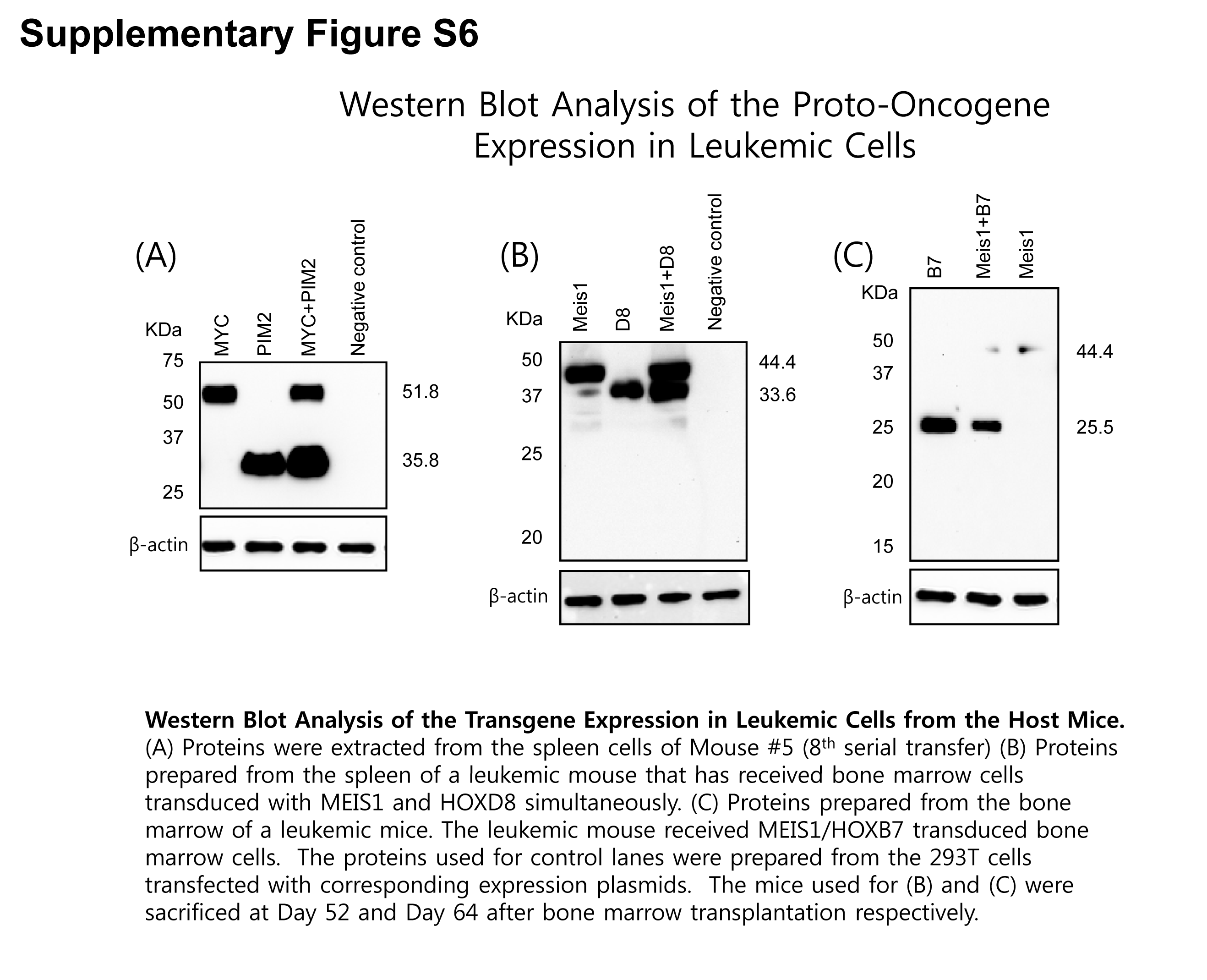

Supplement: S6 Fig — (TIF) [file pone.0143240.s006.tif]
