# Supplementary material for: Characterization of Leukemia-Inducing Genes Using a Proto-Oncogene/Homeobox Gene Retroviral Human cDNA Library in a Mouse In Vivo Model
Source: PLoS One. 2015 Nov 25;10(11):e0143240. doi: 10.1371/journal.pone.0143240 (PMC4659616; doi:10.1371/journal.pone.0143240)
Supplement: S1 Table — (DOCX) [file pone.0143240.s007.docx]

**S1 Table. List of cDNAs included in Retroviral proto-oncogene and homeobox gene cDNA library**

| **Gene Symbol** | **Gene Definition** | **IMAGE Id.** | **NCBI accession** | **CDS [bp]** |
| --- | --- | --- | --- | --- |
| BMI1 | Homo sapiens BMI1 polycomb ring finger oncogene | 4138748 | BC011652 | 981 |
| [CDX1](http://cgap.nci.nih.gov/Genes/RunUniGeneQuery?PAGE=1&ORG=Hs&SYM=&PATH=&TERM=CDX1) | Homo sapiens caudal type homeobox 1, mRNA, complete cds. | 40005955 | BC096252 | 798 |
| CLDN1 | Homo sapiens claudin 1 | 4500534 | BC012471 | 636 |
| [CRK](http://cgap.nci.nih.gov/Genes/RunUniGeneQuery?PAGE=1&ORG=Hs&SYM=&PATH=&TERM=CRK) | v-crk sarcoma virus CT10 oncogene homolog (avian), mRNA, complete cds | 4040536 | BC009837 | 615 |
| CRKL | Homo sapiens v-crk sarcoma virus CT10 oncogene homolog (avian)-like, mRNA | 6046673 | BC043500 | 912 |
| [CRX](http://cgap.nci.nih.gov/Genes/RunUniGeneQuery?PAGE=1&ORG=Hs&SYM=&PATH=&TERM=CRX) | Homo sapiens cone-rod homeobox, mRNA, complete cds | 3869862 | BC016664 | 900 |
| [DEK](http://cgap.nci.nih.gov/Genes/RunUniGeneQuery?PAGE=1&ORG=Hs&SYM=&PATH=&TERM=DEK) | Homo sapiens DEK oncogene (DNA binding), mRNA, complete cds | 5122743 | BC035259 | 1128 |
| [DLX1](http://cgap.nci.nih.gov/Genes/RunUniGeneQuery?PAGE=1&ORG=Hs&SYM=&PATH=&TERM=DLX1) | Homo sapiens distal-less homeobox 1, mRNA, complete cds | 5311747 | BC036189 | 768 |
| [DLX2](http://cgap.nci.nih.gov/Genes/RunUniGeneQuery?PAGE=1&ORG=Hs&SYM=&PATH=&TERM=DLX2) | Homo sapiens distal-less homeobox 2, mRNA, complete cds | 5562689 | BC032558 | 987 |
| [DLX3](http://cgap.nci.nih.gov/Genes/RunUniGeneQuery?PAGE=1&ORG=Hs&SYM=&PATH=&TERM=DLX3) | Homo sapiens distal-less homeobox 3, mRNA, complete cds | 4994099 | BC028970 | 864 |
| [DLX4](http://cgap.nci.nih.gov/Genes/RunUniGeneQuery?PAGE=1&ORG=Hs&SYM=&PATH=&TERM=DLX4) | Homo sapiens distal-less homeobox 4, mRNA, complete cds | 3907376 | BC016145 | 723 |
| [DLX5](http://cgap.nci.nih.gov/Genes/RunUniGeneQuery?PAGE=1&ORG=Hs&SYM=&PATH=&TERM=DLX5) | Homo sapiens distal-less homeobox 5, mRNA, complete cds | 3941691 | BC006226 | 870 |
| [ELK1](http://cgap.nci.nih.gov/Genes/RunUniGeneQuery?PAGE=1&ORG=Hs&SYM=&PATH=&TERM=ELK1) | ELK1, member of ETS oncogene family | 6503270 | BC056150 | 1287 |
| ERBB3 | v-erb-b2 erythroblastic leukemia viral oncogene homolog 3 (avian) | [6147464](http://mgc.nci.nih.gov/Reagents/CloneInfo?ORG=Hs&IMAGE=6147464) | [BC082992](javascript:spawn(%22http://www.ncbi.nlm.nih.gov/entrez/query.fcgi?db=Nucleotide&CMD=Search&term=BC082992%22)) | 4029 |
| ERG | v-ets erythroblastosis virus E26 oncogene homolog (avian) | [6052140](http://mgc.nci.nih.gov/Reagents/CloneInfo?ORG=Hs&IMAGE=6052140) | [BC040168](javascript:spawn(%22http://www.ncbi.nlm.nih.gov/entrez/query.fcgi?db=Nucleotide&CMD=Search&term=BC040168%22)) | 1440 |
| ETAA1 | Ewing tumor-associated antigen 1 | [5556133](http://mgc.nci.nih.gov/Reagents/CloneInfo?ORG=Hs&IMAGE=5556133) | [BC040001](javascript:spawn(%22http://www.ncbi.nlm.nih.gov/entrez/query.fcgi?db=Nucleotide&CMD=Search&term=BC040001%22)) | 2781 |
| ETS1 | v-ets erythroblastosis virus E26 oncogene homolog 1 (avian) | [3946751](http://mgc.nci.nih.gov/Reagents/CloneInfo?ORG=Hs&IMAGE=3946751) | [BC017314](javascript:spawn(%22http://www.ncbi.nlm.nih.gov/entrez/query.fcgi?db=Nucleotide&CMD=Search&term=BC017314%22)) | 819 |
| [ETS2](http://cgap.nci.nih.gov/Genes/RunUniGeneQuery?PAGE=1&ORG=Hs&SYM=&PATH=&TERM=ETS2) | Homo sapiens v-ets erythroblastosis virus E26 oncogene homolog 2 (avian), mRNA, complete cds | 3852274 | BC017040 | 1410 |
| ETV6 | ets variant gene 6 (TEL oncogene) | [6014394](http://mgc.nci.nih.gov/Reagents/CloneInfo?ORG=Hs&IMAGE=6014394) | [BC043399](javascript:spawn(%22http://www.ncbi.nlm.nih.gov/entrez/query.fcgi?db=Nucleotide&CMD=Search&term=BC043399%22)) | 1356 |
| [ETV7](http://cgap.nci.nih.gov/Genes/RunUniGeneQuery?PAGE=1&ORG=Hs&SYM=&PATH=&TERM=ETV7) | Homo sapiens ets variant gene 7 (TEL2 oncogene), mRNA complete cds | 5752352 | BC035853 | 1026 |
| [FES](http://cgap.nci.nih.gov/Genes/RunUniGeneQuery?PAGE=1&ORG=Hs&SYM=&PATH=&TERM=FES) | Homo sapiens feline sarcoma oncogene, mRNA, complete cds | 5170548 | BC035357 | 2469 |
| FEV | FEV (ETS oncogene family) | [4130242](http://mgc.nci.nih.gov/Reagents/CloneInfo?ORG=Hs&IMAGE=4130242) | [BC023511](javascript:spawn(%22http://www.ncbi.nlm.nih.gov/entrez/query.fcgi?db=Nucleotide&CMD=Search&term=BC023511%22)) | 717 |
| FGFR1OP | FGFR1 oncogene partner | [4120239](http://mgc.nci.nih.gov/Reagents/CloneInfo?ORG=Hs&IMAGE=4120239) | [BC011902](javascript:spawn(%22http://www.ncbi.nlm.nih.gov/entrez/query.fcgi?db=Nucleotide&CMD=Search&term=BC011902%22)) | 1140 |
| FGFR1OP2 | FGFR1 oncogene partner 2 | [4552774](http://mgc.nci.nih.gov/Reagents/CloneInfo?ORG=Hs&IMAGE=4552774) | [BC032143](javascript:spawn(%22http://www.ncbi.nlm.nih.gov/entrez/query.fcgi?db=Nucleotide&CMD=Search&term=BC032143%22)) | 519 |
| FGR | Gardner-Rasheed feline sarcoma viral (v-fgr) oncogene homolog | [5734966](http://mgc.nci.nih.gov/Reagents/CloneInfo?ORG=Hs&IMAGE=5734966) | [BC064382](javascript:spawn(%22http://www.ncbi.nlm.nih.gov/entrez/query.fcgi?db=Nucleotide&CMD=Search&term=BC064382%22)) | 1590 |
| [FOS](http://cgap.nci.nih.gov/Genes/RunUniGeneQuery?PAGE=1&ORG=Hs&SYM=&PATH=&TERM=FOS) | Homo sapiens v-fos FBJ murine osteosarcoma viral oncogene homolog, mRNA, complete cds | 3688670 | BC004490 | 1140 |
| FOSB | Homo sapiens FBJ murine osteosarcoma viral oncogene homolog B, mRNA | 5212854 | BC036724 | 1017 |
| [FYN](http://cgap.nci.nih.gov/Genes/RunUniGeneQuery?PAGE=1&ORG=Hs&SYM=&PATH=&TERM=FYN) | Homo sapiens FYN oncogene related to SRC, FGR, YES, mRNA, complete cds | 5219761 | BC032496 | 1614 |
| [GLI](http://cgap.nci.nih.gov/Genes/RunUniGeneQuery?PAGE=1&ORG=Hs&SYM=&PATH=&TERM=GLI) | glioma-associated oncogene homolog 1 (zinc finger protein), mRNA, complete | 3531657 | BC013000 | 3321 |
| [HHEX](http://cgap.nci.nih.gov/Genes/RunUniGeneQuery?PAGE=1&ORG=Hs&SYM=&PATH=&TERM=HHEX) | Homo sapiens hematopoietically expressed homeobox, mRNA, complete cds | 4046641 | BC014336 | 813 |
| [HLX1](http://cgap.nci.nih.gov/Genes/RunUniGeneQuery?PAGE=1&ORG=Hs&SYM=&PATH=&TERM=HLX1) | Homo sapiens H2.0-like homeobox, mRNA, complete cds | 5184776 | BC033808 | 1467 |
| [HOXA1](http://cgap.nci.nih.gov/Genes/RunUniGeneQuery?PAGE=1&ORG=Hs&SYM=&PATH=&TERM=HOXA1) | Homo sapiens homeobox A1, mRNA, complete cds | 5537563 | BC032547 | 1008 |
| [HOXA10](http://cgap.nci.nih.gov/Genes/RunUniGeneQuery?PAGE=1&ORG=Hs&SYM=&PATH=&TERM=HOXA10) | Homo sapiens homeobox A10, mRNA, complete cds | 4107013 | BC013971 | 1182 |
| HOXA11 | Homo sapiens homeobox A11, mRNA, complete cds | [5587615](http://mgc.nci.nih.gov/Reagents/CloneInfo?ORG=Hs&IMAGE=5587615) | [BC040948](javascript:spawn(%22http://www.ncbi.nlm.nih.gov/entrez/query.fcgi?db=Nucleotide&CMD=Search&term=BC040948%22)) | 942 |
| [HOXA3](http://cgap.nci.nih.gov/Genes/RunUniGeneQuery?PAGE=1&ORG=Hs&SYM=&PATH=&TERM=HOXA3) | Homo sapiens homeobox A3, mRNA, complete cds | 3905733 | BC015180 | 1332 |
| [HOXA5](http://cgap.nci.nih.gov/Genes/RunUniGeneQuery?PAGE=1&ORG=Hs&SYM=&PATH=&TERM=HOXA5) | Homo sapiens homeobox A5, mRNA, complete cds | 3863578 | BC013682 | 813 |
| HOXA6 | Homo sapiens homeobox A6, mRNA, complete cds | [8143920](http://mgc.nci.nih.gov/Reagents/CloneInfo?ORG=Hs&IMAGE=8143920) | [BC104917](javascript:spawn(%22http://www.ncbi.nlm.nih.gov/entrez/query.fcgi?db=Nucleotide&CMD=Search&term=BC104917%22)) | 702 |
| [HOXA9](http://cgap.nci.nih.gov/Genes/RunUniGeneQuery?PAGE=1&ORG=Hs&SYM=&PATH=&TERM=HOXA9) | Homo sapiens homeobox A9, mRNA, complete cds | 2987818 | BC010023 | 819 |
| HOXB1 | Homo sapiens homeobox B1, mRNA, complete cds | [40004086](http://mgc.nci.nih.gov/Reagents/CloneInfo?ORG=Hs&IMAGE=40004086) | [BC096193](javascript:spawn(%22http://www.ncbi.nlm.nih.gov/entrez/query.fcgi?db=Nucleotide&CMD=Search&term=BC096193%22)) | 708 |
| HOXB13 | Homo sapiens homeoboxB13, mRNA, complete cds | [6668163](http://mgc.nci.nih.gov/Reagents/CloneInfo?ORG=Hs&IMAGE=6668163) | [BC070233](javascript:spawn(%22http://www.ncbi.nlm.nih.gov/entrez/query.fcgi?db=Nucleotide&CMD=Search&term=BC070233%22)) | 855 |
| HOXB2 | Homo sapiens homeobox B2, mRNA, complete cds | [30915314](http://mgc.nci.nih.gov/Reagents/CloneInfo?ORG=Hs&IMAGE=30915314) | [BC074806](javascript:spawn(%22http://www.ncbi.nlm.nih.gov/entrez/query.fcgi?db=Nucleotide&CMD=Search&term=BC074806%22)) | 1071 |
| HOXB4 | Homo sapiens homeobox B4, mRNA, complete cds | 5533346 | BC049204 | 756 |
| HOXB5 | Homo sapiens homeobox B5, mRNA, complete cds | [40125798](http://mgc.nci.nih.gov/Reagents/CloneInfo?ORG=Hs&IMAGE=40125798) | [BC117247](javascript:spawn(%22http://www.ncbi.nlm.nih.gov/entrez/query.fcgi?db=Nucleotide&CMD=Search&term=BC117247%22)) | 810 |

| **Gene Symbol** | **Gene Definition** | **IMAGE Id.** | **NCBI accession** | **CDS [bp]** |
| --- | --- | --- | --- | --- |
| [HOXB6](http://cgap.nci.nih.gov/Genes/RunUniGeneQuery?PAGE=1&ORG=Hs&SYM=&PATH=&TERM=HOXB6) | Homo sapiens homeobox B6, mRNA, complete cds | 4548382 | BC014651 | 675 |
| [HOXB7](http://cgap.nci.nih.gov/Genes/RunUniGeneQuery?PAGE=1&ORG=Hs&SYM=&PATH=&TERM=HOXB7) | Homo sapiens homeobox B7, mRNA, complete cds | 4413080 | BC015345 | 654 |
| [HOXB9](http://cgap.nci.nih.gov/Genes/RunUniGeneQuery?PAGE=1&ORG=Hs&SYM=&PATH=&TERM=HOXB9) | Homo sapiens homeobox B9, mRNA, complete cds | 4139164 | BC015565 | 753 |
| HOXC10 | homeobox C10 | [3458115](http://mgc.nci.nih.gov/Reagents/CloneInfo?ORG=Hs&IMAGE=3458115) | [BC001293](javascript:spawn(%22http://www.ncbi.nlm.nih.gov/entrez/query.fcgi?db=Nucleotide&CMD=Search&term=BC001293%22)) | 1029 |
| [HOXC11](http://cgap.nci.nih.gov/Genes/RunUniGeneQuery?PAGE=1&ORG=Hs&SYM=&PATH=&TERM=HOXC11) | Homo sapiens homeobox C11, mRNA, complete cds | 3462682 | BC001543 | 915 |
| HOXC13 | homeobox C13 | [6171228](http://mgc.nci.nih.gov/Reagents/CloneInfo?ORG=Hs&IMAGE=6171228) | [BC090850](javascript:spawn(%22http://www.ncbi.nlm.nih.gov/entrez/query.fcgi?db=Nucleotide&CMD=Search&term=BC090850%22)) | 993 |
| HOXC8 | homeobox C8 | [6171090](http://mgc.nci.nih.gov/Reagents/CloneInfo?ORG=Hs&IMAGE=6171090) | [BC053898](javascript:spawn(%22http://www.ncbi.nlm.nih.gov/entrez/query.fcgi?db=Nucleotide&CMD=Search&term=BC053898%22)) | 729 |
| HOXC9 | homeobox C9 | [6144625](http://mgc.nci.nih.gov/Reagents/CloneInfo?ORG=Hs&IMAGE=6144625) | [BC053894](javascript:spawn(%22http://www.ncbi.nlm.nih.gov/entrez/query.fcgi?db=Nucleotide&CMD=Search&term=BC053894%22)) | 783 |
| [HOXD1](http://cgap.nci.nih.gov/Genes/RunUniGeneQuery?PAGE=1&ORG=Hs&SYM=&PATH=&TERM=HOXD1) | Homo sapiens homeobox D1, mRNA, complete cds | 4869019 | BC014477 | 987 |
| HOXD10 | homeobox D10 | [7262455](http://mgc.nci.nih.gov/Reagents/CloneInfo?ORG=Hs&IMAGE=7262455) | [BC069619](javascript:spawn(%22http://www.ncbi.nlm.nih.gov/entrez/query.fcgi?db=Nucleotide&CMD=Search&term=BC069619%22)) | 1023 |
| HOXD12 | homeobox D12 | [40120253](http://mgc.nci.nih.gov/Reagents/CloneInfo?ORG=Hs&IMAGE=40120253) | [BC121104](javascript:spawn(%22http://www.ncbi.nlm.nih.gov/entrez/query.fcgi?db=Nucleotide&CMD=Search&term=BC121104%22)) | 654 |
| [HOXD3](http://cgap.nci.nih.gov/Genes/RunUniGeneQuery?PAGE=1&ORG=Hs&SYM=&PATH=&TERM=HOXD3) | Homo sapiens homeobox D3, mRNA, complete cds | 3936607 | BC005124 | 1299 |
| [HOXD4](http://cgap.nci.nih.gov/Genes/RunUniGeneQuery?PAGE=1&ORG=Hs&SYM=&PATH=&TERM=HOXD4) | Homo sapiens homeobox D4, mRNA, complete cds | 4068219 | BC016763 | 768 |
| HOXD8 | homeobox D8 | [6180525](http://mgc.nci.nih.gov/Reagents/CloneInfo?ORG=Hs&IMAGE=6180525) | [BC090853](javascript:spawn(%22http://www.ncbi.nlm.nih.gov/entrez/query.fcgi?db=Nucleotide&CMD=Search&term=BC090853%22)) | 870 |
| HOXD9 | homeobox D9 | [5191006](http://mgc.nci.nih.gov/Reagents/CloneInfo?ORG=Hs&IMAGE=5191006) | [BC044855](javascript:spawn(%22http://www.ncbi.nlm.nih.gov/entrez/query.fcgi?db=Nucleotide&CMD=Search&term=BC044855%22)) | 1029 |
| [JUN](http://cgap.nci.nih.gov/Genes/RunUniGeneQuery?PAGE=1&ORG=Hs&SYM=&PATH=&TERM=JUN) | jun oncogene, mRNA, complete cds | 4053956 | BC006175 | 996 |
| [JUNB](http://cgap.nci.nih.gov/Genes/RunUniGeneQuery?PAGE=1&ORG=Hs&SYM=&PATH=&TERM=JUNB) | Homo sapiens jun B proto-oncogene, mRNA, complete cds | 3621911 | BC004250 | 1044 |
| [K-RAS2](http://cgap.nci.nih.gov/Genes/RunUniGeneQuery?PAGE=1&ORG=Hs&SYM=&PATH=&TERM=KRAS2) | v-Ki-ras2 Kirsten rat sarcoma viral oncogene homolog, mRNA, complete cds | 3878884 | BC013572 | 567 |
| [LCN2](http://cgap.nci.nih.gov/Genes/RunUniGeneQuery?PAGE=1&ORG=Hs&SYM=&PATH=&TERM=LCN2) | lipocalin 2 | 5421124 | BC033089 | 597 |
| LHX1 | Homo sapiens LIM homeobox 1, mRNA | 8069180 | BC101674 | 1221 |
| LHX2 | LIM homeobox 2 | [7939507](http://mgc.nci.nih.gov/Reagents/CloneInfo?ORG=Hs&IMAGE=7939507) | [BC093662](javascript:spawn(%22http://www.ncbi.nlm.nih.gov/entrez/query.fcgi?db=Nucleotide&CMD=Search&term=BC093662%22)) | 1221 |
| LHX4 | LIM homeobox 4 | [3529089](http://mgc.nci.nih.gov/Reagents/CloneInfo?ORG=Hs&IMAGE=3529089) | [BC011759](javascript:spawn(%22http://www.ncbi.nlm.nih.gov/entrez/query.fcgi?db=Nucleotide&CMD=Search&term=BC011759%22)) | 1173 |
| LHX5 | LIM homeobox 5 | [40010942](http://mgc.nci.nih.gov/Reagents/CloneInfo?ORG=Hs&IMAGE=40010942) | [BC109230](javascript:spawn(%22http://www.ncbi.nlm.nih.gov/entrez/query.fcgi?db=Nucleotide&CMD=Search&term=BC109230%22)) | 1209 |
| LHX6 | LIM homeobox 6 | [40008210](http://mgc.nci.nih.gov/Reagents/CloneInfo?ORG=Hs&IMAGE=40008210) | [BC103937](javascript:spawn(%22http://www.ncbi.nlm.nih.gov/entrez/query.fcgi?db=Nucleotide&CMD=Search&term=BC103937%22)) | 1092 |
| LHX8 | LIM homeobox 8 | [4839343](http://mgc.nci.nih.gov/Reagents/CloneInfo?ORG=Hs&IMAGE=4839343) | [BC040321](javascript:spawn(%22http://www.ncbi.nlm.nih.gov/entrez/query.fcgi?db=Nucleotide&CMD=Search&term=BC040321%22)) | 1071 |
| LHX9 | LIM homeobox 9 | [40117467](http://mgc.nci.nih.gov/Reagents/CloneInfo?ORG=Hs&IMAGE=40117467) | [BC131622](javascript:spawn(%22http://www.ncbi.nlm.nih.gov/entrez/query.fcgi?db=Nucleotide&CMD=Search&term=BC131622%22)) | 1194 |
| LYN | v-yes-1 Yamaguchi sarcoma viral related oncogene homolog | 8992174 | BC126458 | 1539 |
| [MAFB](http://cgap.nci.nih.gov/Genes/RunUniGeneQuery?PAGE=1&ORG=Hs&SYM=&PATH=&TERM=MAFB) | Homo sapiens v-maf musculoaponeurotic fibrosarcoma oncogene homolog B (avian), mRNA | 5261053 | BC036689 | 972 |
| MAFF | v-maf musculoaponeurotic fibrosarcoma oncogene homolog F (avian) | 30335753 | BC067751 | 495 |
| [MAFG](http://cgap.nci.nih.gov/Genes/RunUniGeneQuery?PAGE=1&ORG=Hs&SYM=&PATH=&TERM=MAFG) | v-maf musculoaponeurotic fibrosarcoma oncogene homolog G (avian), mRNA, complete cds | 4637130 | BC012327 | 489 |
| MAS1 | MAS1 oncogene | [7262257](http://mgc.nci.nih.gov/Reagents/CloneInfo?ORG=Hs&IMAGE=7262257) | [BC069581](javascript:spawn(%22http://www.ncbi.nlm.nih.gov/entrez/query.fcgi?db=Nucleotide&CMD=Search&term=BC069581%22)) | 978 |
| MAS1L | MAS1 oncogene-like | [40016384](http://mgc.nci.nih.gov/Reagents/CloneInfo?ORG=Hs&IMAGE=40016384) | [BC101176](javascript:spawn(%22http://www.ncbi.nlm.nih.gov/entrez/query.fcgi?db=Nucleotide&CMD=Search&term=BC101176%22)) | 1137 |
| MEIS1 | Meis homeobox 1 | [5266491](http://mgc.nci.nih.gov/Reagents/CloneInfo?ORG=Hs&IMAGE=5266491) | [BC036511](javascript:spawn(%22http://www.ncbi.nlm.nih.gov/entrez/query.fcgi?db=Nucleotide&CMD=Search&term=BC036511%22)) | 1173 |
| MEIS2 | Meis homeobox 2 | [2964406](http://mgc.nci.nih.gov/Reagents/CloneInfo?ORG=Hs&IMAGE=2964406) | [BC001844](javascript:spawn(%22http://www.ncbi.nlm.nih.gov/entrez/query.fcgi?db=Nucleotide&CMD=Search&term=BC001844%22)) | 1146 |
| [MEOX2](http://cgap.nci.nih.gov/Genes/RunUniGeneQuery?PAGE=1&ORG=Hs&SYM=&PATH=&TERM=MEOX2) | Homo sapiens mesenchyme homeobox 2, mRNA, complete cds | 3917118 | BC017021 | 912 |
| MOS | v-mos Moloney murine sarcoma viral oncogene homolog | [40016106](http://mgc.nci.nih.gov/Reagents/CloneInfo?ORG=Hs&IMAGE=40016106) | [BC106738](javascript:spawn(%22http://www.ncbi.nlm.nih.gov/entrez/query.fcgi?db=Nucleotide&CMD=Search&term=BC106738%22)) | 1041 |
| MSH2 | Homo sapiens mutS homolog 2 | 4110354 | BC021566 | 2805 |
| [MSX2](http://cgap.nci.nih.gov/Genes/RunUniGeneQuery?PAGE=1&ORG=Hs&SYM=&PATH=&TERM=MSX2) | Homo sapiens msh homeobox 2, mRNA, complete cds | 3903028 | BC015509 | 804 |
| MYB | v-myb myeloblastosis viral oncogene homolog (avian) | [6069320](http://mgc.nci.nih.gov/Reagents/CloneInfo?ORG=Hs&IMAGE=6069320) | [BC064955](javascript:spawn(%22http://www.ncbi.nlm.nih.gov/entrez/query.fcgi?db=Nucleotide&CMD=Search&term=BC064955%22)) | 1923 |
| MYBL1 | v-myb myeloblastosis viral oncogene homolog (avian)-like 1 | [40018967](http://mgc.nci.nih.gov/Reagents/CloneInfo?ORG=Hs&IMAGE=40018967) | [BC101186](javascript:spawn(%22http://www.ncbi.nlm.nih.gov/entrez/query.fcgi?db=Nucleotide&CMD=Search&term=BC101186%22)) | 2076 |
| [MYBL2](http://cgap.nci.nih.gov/Genes/RunUniGeneQuery?PAGE=1&ORG=Hs&SYM=&PATH=&TERM=MYBL2) | v-myb myeloblastosis viral oncogene homolog (avian)-like 2, mRNA, complete cds | 3162656 | BC007585 | 2103 |
| [MYC](http://cgap.nci.nih.gov/Genes/RunUniGeneQuery?PAGE=1&ORG=Hs&SYM=&PATH=&TERM=MYC) | Homo sapiens v-myc myelocytomatosis viral oncogene homolog (avian), mRNA, complete cds | 2985844 | BC000141 | 1365 |
| [MYCL1](http://cgap.nci.nih.gov/Genes/RunUniGeneQuery?PAGE=1&ORG=Hs&SYM=&PATH=&TERM=MYCL1) | v-myc myelocytomatosis viral oncogene homolog 1, lung carcinoma derived (avian), mRNA, complete cds | 4541675 | BC011864 | 621 |
| MYCNOS | v-myc myelocytomatosis viral related oncogene, neuroblastoma derived (avian) opposite strand | 3940152 | NM_006316 | 330 |
| [N-RAS](http://cgap.nci.nih.gov/Genes/RunUniGeneQuery?PAGE=1&ORG=Hs&SYM=&PATH=&TERM=NRAS) | Homo sapiens neuroblastoma RAS viral (v-ras) oncogene homolog, mRNA, complete cds | 3826638 | BC005219 | 570 |

| **Gene Symbol** | **Gene Definition** | **IMAGE Id.** | **NCBI accession** | **CDS [bp]** |
| --- | --- | --- | --- | --- |
| PBX3 | pre-B-cell leukemia homeobox 3 | [5212090](http://mgc.nci.nih.gov/Reagents/CloneInfo?ORG=Hs&IMAGE=5212090) | [BC094883](javascript:spawn(%22http://www.ncbi.nlm.nih.gov/entrez/query.fcgi?db=Nucleotide&CMD=Search&term=BC094883%22)) | 1302 |
| PBX4 | pre-B-cell leukemia homeobox 4 | [8860384](http://mgc.nci.nih.gov/Reagents/CloneInfo?ORG=Hs&IMAGE=8860384) | [BC141859](javascript:spawn(%22http://www.ncbi.nlm.nih.gov/entrez/query.fcgi?db=Nucleotide&CMD=Search&term=BC141859%22)) | 1125 |
| PBXIP1 | pre-B-cell leukemia homeobox interacting protein 1 | [3900461](http://mgc.nci.nih.gov/Reagents/CloneInfo?ORG=Hs&IMAGE=3900461) | [BC016852](javascript:spawn(%22http://www.ncbi.nlm.nih.gov/entrez/query.fcgi?db=Nucleotide&CMD=Search&term=BC016852%22)) | 2196 |
| [PDGFB](http://cgap.nci.nih.gov/Genes/RunUniGeneQuery?PAGE=1&ORG=Hs&SYM=&PATH=&TERM=PDGFB) | Homo sapiens platelet-derived growth factor beta polypeptide (simian sarcoma viral (v-sis) oncogene homolog), mRNA , complete cds | 5174750 | BC029822 | 726 |
| [PIM1](http://cgap.nci.nih.gov/Genes/RunUniGeneQuery?PAGE=1&ORG=Hs&SYM=&PATH=&TERM=PIM1) | Proto-oncogene serine/threonine-protein kinase Pim-1, mRNA, complete cds | 4591723 | BC020224 | 942 |
| [PIM2](http://cgap.nci.nih.gov/Genes/RunUniGeneQuery?PAGE=1&ORG=Hs&SYM=&PATH=&TERM=PIM2) | Homo sapiens pim-2 oncogene, mRNA, complete cds | 3913552 | BC018111 | 936 |
| PIM3 | pim-3 oncogene | [8860375](http://mgc.nci.nih.gov/Reagents/CloneInfo?ORG=Hs&IMAGE=8860375) | [BC141855](javascript:spawn(%22http://www.ncbi.nlm.nih.gov/entrez/query.fcgi?db=Nucleotide&CMD=Search&term=BC141855%22)) | 981 |
| POU2F2 | POU class 2 homeobox 2 | [3688152](http://mgc.nci.nih.gov/Reagents/CloneInfo?ORG=Hs&IMAGE=3688152) | [BC006101](javascript:spawn(%22http://www.ncbi.nlm.nih.gov/entrez/query.fcgi?db=Nucleotide&CMD=Search&term=BC006101%22)) | 1203 |
| POU2F3 | POU class 2 homeobox 3 | [8069155](http://mgc.nci.nih.gov/Reagents/CloneInfo?ORG=Hs&IMAGE=8069155) | [BC101649](javascript:spawn(%22http://www.ncbi.nlm.nih.gov/entrez/query.fcgi?db=Nucleotide&CMD=Search&term=BC101649%22)) | 1311 |
| POU4F3 | POU class 4 homeobox 3 | [8143926](http://mgc.nci.nih.gov/Reagents/CloneInfo?ORG=Hs&IMAGE=8143926) | [BC104923](javascript:spawn(%22http://www.ncbi.nlm.nih.gov/entrez/query.fcgi?db=Nucleotide&CMD=Search&term=BC104923%22)) | 1017 |
| POU5F1 | POU class 5 homeobox 1 | [40125988](http://mgc.nci.nih.gov/Reagents/CloneInfo?ORG=Hs&IMAGE=40125988) | [BC117437](javascript:spawn(%22http://www.ncbi.nlm.nih.gov/entrez/query.fcgi?db=Nucleotide&CMD=Search&term=BC117437%22)) | 1083 |
| POU6F1 | POU class 6 homeobox 1 | [6275848](http://mgc.nci.nih.gov/Reagents/CloneInfo?ORG=Hs&IMAGE=6275848) | [BC051326](javascript:spawn(%22http://www.ncbi.nlm.nih.gov/entrez/query.fcgi?db=Nucleotide&CMD=Search&term=BC051326%22)) | 906 |
| [PROX1](http://cgap.nci.nih.gov/Genes/RunUniGeneQuery?PAGE=1&ORG=Hs&SYM=&PATH=&TERM=PROX1) | Homo sapiens prospero homeobox 1, mRNA, complete cds | 3532312 | BC024201 | 2214 |
| PTTG1 | Homo sapiens pituitary tumor-transforming 1 | 40113885 | BC128193 | 609 |
| [RAB10](http://cgap.nci.nih.gov/Genes/RunUniGeneQuery?PAGE=1&ORG=Hs&SYM=&PATH=&TERM=RAB10) | Homo sapiens RAB10, member RAS oncogene family, mRNA, complete cds | 3464547 | BC000896 | 603 |
| [RAB11A](http://cgap.nci.nih.gov/Genes/RunUniGeneQuery?PAGE=1&ORG=Hs&SYM=&PATH=&TERM=RAB11A) | Rab11a GTPase, member RAS oncogene family | 3510339 | BC013348 | 651 |
| [RAB13](http://cgap.nci.nih.gov/Genes/RunUniGeneQuery?PAGE=1&ORG=Hs&SYM=&PATH=&TERM=RAB13) | Homo sapiens RAB13, member RAS oncogene family, mRNA, complete cds | 3451945 | BC000799 | 612 |
| [RAB14](http://cgap.nci.nih.gov/Genes/RunUniGeneQuery?PAGE=1&ORG=Hs&SYM=&PATH=&TERM=RAB14) | Homo sapiens RAB14, member RAS oncogene family, mRNA, complete cds | 2963119 | BC006081 | 648 |
| [RAB17](http://cgap.nci.nih.gov/Genes/RunUniGeneQuery?PAGE=1&ORG=Hs&SYM=&PATH=&TERM=RAB17) | RAB17, member RAS oncogene family, mRNA, complete cds | 5761178 | BC050426 | 639 |
| [RAB18](http://cgap.nci.nih.gov/Genes/RunUniGeneQuery?PAGE=1&ORG=Hs&SYM=&PATH=&TERM=RAB18) | Homo sapiens RAB18, member RAS oncogene family, mRNA, complete cds | 3922029 | BC015014 | 621 |
| [RAB1A](http://cgap.nci.nih.gov/Genes/RunUniGeneQuery?PAGE=1&ORG=Hs&SYM=&PATH=&TERM=RAB1A) | RAB1A, member RAS oncogene family, mRNA , complete cds | 2900705 | BC000905 | 618 |
| [RAB1B](http://cgap.nci.nih.gov/Genes/RunUniGeneQuery?PAGE=1&ORG=Hs&SYM=&PATH=&TERM=RAB1B) | RAB1B, member RAS oncogene family, mRNA, complete cds | 6026121 | BC071169 | 606 |
| [RAB2](http://cgap.nci.nih.gov/Genes/RunUniGeneQuery?PAGE=1&ORG=Hs&SYM=&PATH=&TERM=RAB2) | RAB2A, member RAS oncogene family | 2966694 | BC008929 | 639 |
| [RAB22A](http://cgap.nci.nih.gov/Genes/RunUniGeneQuery?PAGE=1&ORG=Hs&SYM=&PATH=&TERM=RAB22A) | RAB22A, member RAS oncogene family, mRNA, complete cds | 3907891 | BC015710 | 585 |
| [RAB23](http://cgap.nci.nih.gov/Genes/RunUniGeneQuery?PAGE=1&ORG=Hs&SYM=&PATH=&TERM=RAB23) | RAB23, member RAS oncogene family, mRNA, complete cds | 3910708 | BC015021 | 714 |
| [RAB25](http://cgap.nci.nih.gov/Genes/RunUniGeneQuery?PAGE=1&ORG=Hs&SYM=&PATH=&TERM=RAB25) | RAB25, member RAS oncogene family, mRNA, complete cds | 3926839 | BC009831 | 771 |
| [RAB27B](http://cgap.nci.nih.gov/Genes/RunUniGeneQuery?PAGE=1&ORG=Hs&SYM=&PATH=&TERM=RAB27B) | Homo sapiens RAB27B, member RAS oncogene family, mRNA), complete cds | 4520182 | BC027474 | 657 |
| [RAB2A](http://cgap.nci.nih.gov/Genes/RunUniGeneQuery?PAGE=1&ORG=Hs&SYM=&PATH=&TERM=RAB2) | Homo sapiens RAB2A, member RAS oncogene family, mRNA, complete cds | 2966694 | BC008929 | 666 |
| [RAB2B](http://cgap.nci.nih.gov/Genes/RunUniGeneQuery?PAGE=1&ORG=Hs&SYM=&PATH=&TERM=RAB2B) | RAB2B, member RAS oncogene family, mRNA, complete cds | 4306410 | BC020839 | 651 |
| [RAB30](http://cgap.nci.nih.gov/Genes/RunUniGeneQuery?PAGE=1&ORG=Hs&SYM=&PATH=&TERM=RAB30) | RAB30, member RAS oncogene family, mRNA, complete cds | 4763140 | BC014213 | 612 |
| [RAB31](http://cgap.nci.nih.gov/Genes/RunUniGeneQuery?PAGE=1&ORG=Hs&SYM=&PATH=&TERM=RAB31) | Homo sapiens RAB31, member RAS oncogene family, mRNA, complete cds | 3534853 | BC001148 | 585 |
| [RAB33A](http://cgap.nci.nih.gov/Genes/RunUniGeneQuery?PAGE=1&ORG=Hs&SYM=&PATH=&TERM=RAB33A) | RAB33A, member RAS oncogene family, mRNA, complete cds | 4129324 | BC009996 | 714 |
| [RAB34](http://cgap.nci.nih.gov/Genes/RunUniGeneQuery?PAGE=1&ORG=Hs&SYM=&PATH=&TERM=RAB34) | RAB34, member RAS oncogene family, mRNA, complete cds | 3902651 | BC016841 | 780 |
| [RAB35](http://cgap.nci.nih.gov/Genes/RunUniGeneQuery?PAGE=1&ORG=Hs&SYM=&PATH=&TERM=RAB35) | Homo sapiens RAB35, member RAS oncogene family, mRNA, complete cds | 3907209 | BC015931 | 606 |
| [RAB37](http://cgap.nci.nih.gov/Genes/RunUniGeneQuery?PAGE=1&ORG=Hs&SYM=&PATH=&TERM=RAB37) | Homo sapiens RAB37, member RAS oncogene family, mRNA, complete cds | 4520191 | BC016615 | 651 |

| **Gene Symbol** | **Gene Definition** | **IMAGE Id.** | **NCBI accession** | **CDS [bp]** |
| --- | --- | --- | --- | --- |
| [RAB38](http://cgap.nci.nih.gov/Genes/RunUniGeneQuery?PAGE=1&ORG=Hs&SYM=&PATH=&TERM=RAB38) | Homo sapiens RAB38, member RAS oncogene family, mRNA, complete cds | 3916084 | BC015808 | 636 |
| [RAB39](http://cgap.nci.nih.gov/Genes/RunUniGeneQuery?PAGE=1&ORG=Hs&SYM=&PATH=&TERM=RAB39) | Homo sapiens RAB39, member RAS oncogene family, mRNA, complete cds | 5197104 | BC028064 | 654 |
| [RAB3A](http://cgap.nci.nih.gov/Genes/RunUniGeneQuery?PAGE=1&ORG=Hs&SYM=&PATH=&TERM=RAB3A) | RAB3A, member RAS oncogene family, mRNA, complete cds | 3939176 | BC011782 | 663 |
| [RAB3B](http://cgap.nci.nih.gov/Genes/RunUniGeneQuery?PAGE=1&ORG=Hs&SYM=&PATH=&TERM=RAB3B) | Homo sapiens RAB3B, member RAS oncogene family, mRNA, complete cds | 4040566 | BC005035 | 660 |
| [RAB3C](http://cgap.nci.nih.gov/Genes/RunUniGeneQuery?PAGE=1&ORG=Hs&SYM=&PATH=&TERM=RAB3C) | Homo sapiens RAB3C, member RAS oncogene family, mRNA, complete cds | 3534915 | BC013033 | 684 |
| [RAB3D](http://cgap.nci.nih.gov/Genes/RunUniGeneQuery?PAGE=1&ORG=Hs&SYM=&PATH=&TERM=RAB3D) | Homo sapiens RAB3D, member RAS oncogene family, mRNA, complete cds | 3861912 | BC016471 | 660 |
| [RAB40B](http://cgap.nci.nih.gov/Genes/RunUniGeneQuery?PAGE=1&ORG=Hs&SYM=&PATH=&TERM=RAB40B) | Homo sapiens RAB40B, member RAS oncogene family, mRNA, complete cds | 4801271 | BC018039 | 837 |
| [RAB4A](http://cgap.nci.nih.gov/Genes/RunUniGeneQuery?PAGE=1&ORG=Hs&SYM=&PATH=&TERM=RAB4A) | RAB4A, member RAS oncogene family, mRNA, complete cds | 3346455 | BC002438 | 657 |
| [RAB5A](http://cgap.nci.nih.gov/Genes/RunUniGeneQuery?PAGE=1&ORG=Hs&SYM=&PATH=&TERM=RAB5A) | Homo sapiens RAB5A, member RAS oncogene family, mRNA, complete cds | 3463669 | BC001267 | 648 |
| [RAB5C](http://cgap.nci.nih.gov/Genes/RunUniGeneQuery?PAGE=1&ORG=Hs&SYM=&PATH=&TERM=RAB5C) | Homo sapiens RAB5C, member RAS oncogene family, transcript variant 2, mRNA | 4938169 | BC106039 | 651 |
| [RAB6A](http://cgap.nci.nih.gov/Genes/RunUniGeneQuery?PAGE=1&ORG=Hs&SYM=&PATH=&TERM=RAB6A) | Homo sapiens RAB6A, member RAS oncogene family, mRNA, complete cds | 3506585 | BC003617 | 627 |
| [RAB6B](http://cgap.nci.nih.gov/Genes/RunUniGeneQuery?PAGE=1&ORG=Hs&SYM=&PATH=&TERM=RAB6B) | Homo sapiens RAB6B, member RAS oncogene family, mRNA, complete cds | 3050592 | NM_016577 | 627 |
| [RAB7B](http://cgap.nci.nih.gov/Genes/RunUniGeneQuery?PAGE=1&ORG=Hs&SYM=&PATH=&TERM=MGC9726) | Homo sapiens RAB7B, member RAS oncogene family, mRNA, complete cds | 3851998 | BC017092 | 600 |
| [RAB7L1](http://cgap.nci.nih.gov/Genes/RunUniGeneQuery?PAGE=1&ORG=Hs&SYM=&PATH=&TERM=RAB7L1) | Homo sapiens RAB7, member RAS oncogene family-like 1, mRNA, complete cds | 3140744 | BC002585 | 612 |
| [RAB8A](http://cgap.nci.nih.gov/Genes/RunUniGeneQuery?PAGE=1&ORG=Hs&SYM=&PATH=&TERM=RAB8A) | Homo sapiens RAB8A, member RAS oncogene family, mRNA, complete cds | 3547214 | BC002977 | 624 |
| [RAB8B](http://cgap.nci.nih.gov/Genes/RunUniGeneQuery?PAGE=1&ORG=Hs&SYM=&PATH=&TERM=RAB8B) | RAB8B, member RAS oncogene family, mRNA, complete cds | 4701429 | BC020654 | 624 |
| [RAB9A](http://cgap.nci.nih.gov/Genes/RunUniGeneQuery?PAGE=1&ORG=Hs&SYM=&PATH=&TERM=RAB9A) | RAB9A, member RAS oncogene family, mRNA, complete cds | 4139714 | BC017265 | 606 |
| [RABL2B](http://cgap.nci.nih.gov/Genes/RunUniGeneQuery?PAGE=1&ORG=Hs&SYM=&PATH=&TERM=RABL2B) | RAB, member of RAS oncogene family-like 2B, mRNA, complete cds | 4580599 | BC024281 | 687 |
| [RABL3](http://cgap.nci.nih.gov/Genes/RunUniGeneQuery?PAGE=1&ORG=Hs&SYM=&PATH=&TERM=RABL3) | RAB, member of RAS oncogene family-like 3, mRNA, complete cds | 4771781 | BC020832 | 711 |
| [RABL4](http://cgap.nci.nih.gov/Genes/RunUniGeneQuery?PAGE=1&ORG=Hs&SYM=&PATH=&TERM=RABL4) | Homo sapiens RAB, member of RAS oncogene family-like 4, mRNA, complete cds | 3163341 | BC000566 | 558 |
| [RABL5](http://cgap.nci.nih.gov/Genes/RunUniGeneQuery?PAGE=1&ORG=Hs&SYM=&PATH=&TERM=RABL5) | RAB, member RAS oncogene family-like 5, mRNA, complete cds | 3939191 | BC004522 | 558 |
| [RAF1](http://cgap.nci.nih.gov/Genes/RunUniGeneQuery?PAGE=1&ORG=Hs&SYM=&PATH=&TERM=RAF1) | Homo sapiens v-raf-1 murine leukemia viral oncogene homolog 1, mRNA, complete cds | 3904404 | BC018119 | 1947 |
| [RALA](http://cgap.nci.nih.gov/Genes/RunUniGeneQuery?PAGE=1&ORG=Hs&SYM=&PATH=&TERM=RALA) | v-ral simian leukemia viral oncogene homolog A (ras related), mRNA, complete cds | 5495399 | BC039858 | 621 |
| [RALB](http://cgap.nci.nih.gov/Genes/RunUniGeneQuery?PAGE=1&ORG=Hs&SYM=&PATH=&TERM=RALB) | v-ral simian leukemia viral oncogene homolog B (ras related; GTP binding protein), mRNA, complete cds | 3880116 | BC018163 | 621 |
| [RAN](http://cgap.nci.nih.gov/Genes/RunUniGeneQuery?PAGE=1&ORG=Hs&SYM=&PATH=&TERM=RAN) | RAN, member RAS oncogene family, mRNA, complete cds | 3830061 | BC014518 | 651 |
| [RAP1A](http://cgap.nci.nih.gov/Genes/RunUniGeneQuery?PAGE=1&ORG=Hs&SYM=&PATH=&TERM=RAP1A) | RAP1A, member of RAS oncogene family, mRNA, complete cds | 4422971 | BC014086 | 555 |
| [RAP1B](http://cgap.nci.nih.gov/Genes/RunUniGeneQuery?PAGE=1&ORG=Hs&SYM=&PATH=&TERM=RAP1B) | Homo sapiens RAP1B, member of RAS oncogene family, mRNA, complete cds | 2900837 | BC000176 | 555 |
| [RAP2B](http://cgap.nci.nih.gov/Genes/RunUniGeneQuery?PAGE=1&ORG=Hs&SYM=&PATH=&TERM=RAP2B) | RAP2B, member of RAS oncogene family, mRNA, complete cds | 4650072 | BC012362 | 552 |
| [RAP2C](http://cgap.nci.nih.gov/Genes/RunUniGeneQuery?PAGE=1&ORG=Hs&SYM=&PATH=&TERM=RAP2C) | Homo sapiens RAP2C, member of RAS oncogene family, mRNA, complete cds | 3449262 | BC003403 | 552 |
| [RELB](http://cgap.nci.nih.gov/Genes/RunUniGeneQuery?PAGE=1&ORG=Hs&SYM=&PATH=&TERM=RELB) | Homo sapiens v-rel reticuloendotheliosis viral oncogene homolog B, nuclear factor of kappa light polypeptide gene enhancer in B-cells 3 (avian), mRNA, complete cds | 5215944 | BC028013 | 1740 |

| **Gene Symbol** | **Gene Definition** | **IMAGE Id.** | **NCBI accession** | **CDS [bp]** |
| --- | --- | --- | --- | --- |
| RET | ret proto-oncogene | [3160389](http://mgc.nci.nih.gov/Reagents/CloneInfo?ORG=Hs&IMAGE=3160389) | [BC004257](javascript:spawn(%22http://www.ncbi.nlm.nih.gov/entrez/query.fcgi?db=Nucleotide&CMD=Search&term=BC004257%22)) | 3219 |
| [RRAS](http://cgap.nci.nih.gov/Genes/RunUniGeneQuery?PAGE=1&ORG=Hs&SYM=&PATH=&TERM=RRAS) | related RAS viral (r-ras) oncogene homolog, mRNA, complete cds | 4073829 | BC016318 | 657 |
| [RRAS2](http://cgap.nci.nih.gov/Genes/RunUniGeneQuery?PAGE=1&ORG=Hs&SYM=&PATH=&TERM=RRAS2) | Homo sapiens related RAS viral (r-ras) oncogene homolog 2, mRNA, complete cds | 4389214 | BC013106 | 615 |
| SET | SET nuclear oncogene | [5587291](http://mgc.nci.nih.gov/Reagents/CloneInfo?ORG=Hs&IMAGE=5587291) | [BC032749](javascript:spawn(%22http://www.ncbi.nlm.nih.gov/entrez/query.fcgi?db=Nucleotide&CMD=Search&term=BC032749%22)) | 834 |
| [SIX1](http://cgap.nci.nih.gov/Genes/RunUniGeneQuery?PAGE=1&ORG=Hs&SYM=&PATH=&TERM=SIX1) | Homo sapiens SIX homeobox 1, mRNA, complete cds | 4138234 | BC008874 | 855 |
| [SPI1](http://cgap.nci.nih.gov/Genes/RunUniGeneQuery?PAGE=1&ORG=Hs&SYM=&PATH=&TERM=SPI1) | spleen focus forming virus (SFFV) proviral integration oncogene spi1 | 40080653 | BC111379 | 795 |
| SSPN | sarcospan (Kras oncogene-associated gene) | [6376868](http://mgc.nci.nih.gov/Reagents/CloneInfo?ORG=Hs&IMAGE=6376868) | [BC062299](javascript:spawn(%22http://www.ncbi.nlm.nih.gov/entrez/query.fcgi?db=Nucleotide&CMD=Search&term=BC062299%22)) | 732 |
| TACSTD1 | tumor-associated calcium signal transducer 1 | [3861826](http://mgc.nci.nih.gov/Reagents/CloneInfo?ORG=Hs&IMAGE=3861826) | [BC014785](javascript:spawn(%22http://www.ncbi.nlm.nih.gov/entrez/query.fcgi?db=Nucleotide&CMD=Search&term=BC014785%22)) | 945 |
| TACSTD2 | tumor-associated calcium signal transducer 2 | [3611756](http://mgc.nci.nih.gov/Reagents/CloneInfo?ORG=Hs&IMAGE=3611756) | [BC009409](javascript:spawn(%22http://www.ncbi.nlm.nih.gov/entrez/query.fcgi?db=Nucleotide&CMD=Search&term=BC009409%22)) | 972 |
| [TGIF1](http://cgap.nci.nih.gov/Genes/RunUniGeneQuery?PAGE=1&ORG=Hs&SYM=&PATH=&TERM=TGIF) | Homo sapiens TGFB-induced factor homeobox 1, mRNA), complete cds | 3453337 | BC000814 | 759 |
| [TGIF2](http://cgap.nci.nih.gov/Genes/RunUniGeneQuery?PAGE=1&ORG=Hs&SYM=&PATH=&TERM=TGIF2) | Homo sapiens TGFB-induced factor homeobox 2, mRNA, complete cds | 2964507 | BC012816 | 714 |
| [THRA](http://cgap.nci.nih.gov/Genes/RunUniGeneQuery?PAGE=1&ORG=Hs&SYM=&PATH=&TERM=THRA) | thyroid hormone receptor, alpha (erythroblastic leukemia viral (v-erb-a) oncogene homolog, avian), mRNA, complete cds | 3501886 | BC000261 | 1473 |
| THRB | thyroid hormone receptor, beta (erythroblastic leukemia viral (v-erb-a) oncogene homolog 2, avian) | [40033200](http://mgc.nci.nih.gov/Reagents/CloneInfo?ORG=Hs&IMAGE=40033200) | [BC106930](javascript:spawn(%22http://www.ncbi.nlm.nih.gov/entrez/query.fcgi?db=Nucleotide&CMD=Search&term=BC106930%22)) | 1386 |
| [TIMP1](http://cgap.nci.nih.gov/Genes/RunUniGeneQuery?PAGE=1&ORG=Hs&SYM=&PATH=&TERM=TIMP1) | Homo sapiens v-raf murine sarcoma 3611 viral oncogene homolog, mRNA, complete cds | 3347509 | BC002466 | 1821 |
| TLX1 | T-cell leukemia homeobox 1 | [40146561](http://mgc.nci.nih.gov/Reagents/CloneInfo?ORG=Hs&IMAGE=40146561) | [BC130530](javascript:spawn(%22http://www.ncbi.nlm.nih.gov/entrez/query.fcgi?db=Nucleotide&CMD=Search&term=BC130530%22)) | 774 |
| TLX2 | T-cell leukemia homeobox 2 | [4125397](http://mgc.nci.nih.gov/Reagents/CloneInfo?ORG=Hs&IMAGE=4125397) | [BC006356](javascript:spawn(%22http://www.ncbi.nlm.nih.gov/entrez/query.fcgi?db=Nucleotide&CMD=Search&term=BC006356%22)) | 855 |
| TLX3 | T-cell leukemia homeobox 3 | [4906239](http://mgc.nci.nih.gov/Reagents/CloneInfo?ORG=Hs&IMAGE=4906239) | [BC017291](javascript:spawn(%22http://www.ncbi.nlm.nih.gov/entrez/query.fcgi?db=Nucleotide&CMD=Search&term=BC017291%22)) | 876 |
| TMEM205 | Homo sapiens transmembrane protein 205, complete cds | 6025162 | BC064948 | 570 |
| USP4 | ubiquitin specific peptidase 4 (proto-oncogene) | [40119090](http://mgc.nci.nih.gov/Reagents/CloneInfo?ORG=Hs&IMAGE=40119090) | [BC125130](javascript:spawn(%22http://www.ncbi.nlm.nih.gov/entrez/query.fcgi?db=Nucleotide&CMD=Search&term=BC125130%22)) | 2889 |
| [VAX2](http://cgap.nci.nih.gov/Genes/RunUniGeneQuery?PAGE=1&ORG=Hs&SYM=&PATH=&TERM=VAX2) | Homo sapiens ventral anterior homeobox 2, mRNA, complete cds | 4304066 | BC006336 | 873 |
| WT1 | Wilms tumor 1 | [4827849](http://mgc.nci.nih.gov/Reagents/CloneInfo?ORG=Hs&IMAGE=4827849) | [BC032861](javascript:spawn(%22http://www.ncbi.nlm.nih.gov/entrez/query.fcgi?db=Nucleotide&CMD=Search&term=BC032861%22)) | 909 |
| WTAP | Wilms tumor 1 associated protein | [4653831](http://mgc.nci.nih.gov/Reagents/CloneInfo?ORG=Hs&IMAGE=4653831) | [BC069192](javascript:spawn(%22http://www.ncbi.nlm.nih.gov/entrez/query.fcgi?db=Nucleotide&CMD=Search&term=BC069192%22)) | 1191 |
| YES1 | v-yes-1 Yamaguchi sarcoma viral oncogene homolog 1 | [5260751](http://mgc.nci.nih.gov/Reagents/CloneInfo?ORG=Hs&IMAGE=5260751) | [BC048960](javascript:spawn(%22http://www.ncbi.nlm.nih.gov/entrez/query.fcgi?db=Nucleotide&CMD=Search&term=BC048960%22)) | 1632 |
