# Supplementary material for: Characterization of Leukemia-Inducing Genes Using a Proto-Oncogene/Homeobox Gene Retroviral Human cDNA Library in a Mouse In Vivo Model
Source: PLoS One. 2015 Nov 25;10(11):e0143240. doi: 10.1371/journal.pone.0143240 (PMC4659616; doi:10.1371/journal.pone.0143240)
Supplement: S2 Table — (DOCX) [file pone.0143240.s008.docx]

**S2 Table. List of deleted MYC-related proto-oncogenes**

| MYB | v-myb myeloblastosis viral oncogene homolog (avian) | [6069320](http://mgc.nci.nih.gov/Reagents/CloneInfo?ORG=Hs&IMAGE=6069320) | [BC064955](javascript:spawn(%22http://www.ncbi.nlm.nih.gov/entrez/query.fcgi?db=Nucleotide&CMD=Search&term=BC064955%22)) |
| --- | --- | --- | --- |
| MYBL1 | v-myb myeloblastosis viral oncogene homolog (avian)-like 1 | [40018967](http://mgc.nci.nih.gov/Reagents/CloneInfo?ORG=Hs&IMAGE=40018967) | [BC101186](javascript:spawn(%22http://www.ncbi.nlm.nih.gov/entrez/query.fcgi?db=Nucleotide&CMD=Search&term=BC101186%22)) |
| [MYBL2](http://cgap.nci.nih.gov/Genes/RunUniGeneQuery?PAGE=1&ORG=Hs&SYM=&PATH=&TERM=MYBL2) | v-myb myeloblastosis viral oncogene homolog (avian)-like 2, mRNA, complete cds | 3162656 | BC007585 |
| [MYC](http://cgap.nci.nih.gov/Genes/RunUniGeneQuery?PAGE=1&ORG=Hs&SYM=&PATH=&TERM=MYC) | Homo sapiens v-myc myelocytomatosis viral oncogene homolog (avian), mRNA, complete cds | 2985844 | BC000141 |
| [MYCL1](http://cgap.nci.nih.gov/Genes/RunUniGeneQuery?PAGE=1&ORG=Hs&SYM=&PATH=&TERM=MYCL1) | v-myc myelocytomatosis viral oncogene homolog 1, lung carcinoma derived (avian), mRNA, complete cds | 4541675 | BC011864 |
| MYCNOS | v-myc myelocytomatosis viral related oncogene, neuroblastoma derived (avian) opposite strand | 3940152 | NM_006316 |
