# Supplementary material for: Characterization of Leukemia-Inducing Genes Using a Proto-Oncogene/Homeobox Gene Retroviral Human cDNA Library in a Mouse In Vivo Model
Source: PLoS One. 2015 Nov 25;10(11):e0143240. doi: 10.1371/journal.pone.0143240 (PMC4659616; doi:10.1371/journal.pone.0143240)
Supplement: S3 Table — (DOCX) [file pone.0143240.s009.docx]

**
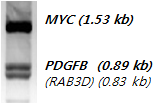
S3 Table. Genomic DNA-PCR_ Sequencing profile**

**MOUSE #1 (*MYC, PDGFb, RAB3D*)**

**Band 1) *MYC***

Cloning site: EcoRI; GAATTC

**Band 2)** ***PDGFb***

**Band 3)** ***RAB3D***

**
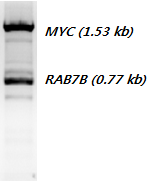
MOUSE #2 (*MYC, RAB7B***

**Band 1) *MYC***

**Band 2) *RAB7B***

**MOUSE #3 (*MYC alone)***


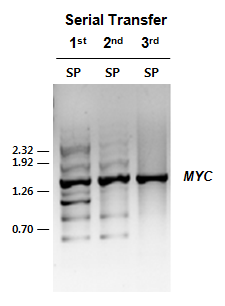


BAND 1

BAND 2

BAND 3

**Band 1**

**Example of Minor Bands Sequencing Failure**

**Band 2**

**Band 3**

**
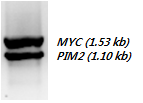
MOUSE #4 (*MYC, PIM2)***

**Band 1) MYC**

**Band 2) PIM2**

**
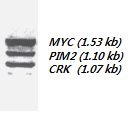
MOUSE #5 (*MYC, PIM2,CRK)***

**Band 1) *MYC***

**Band 2) *PIM2***

**Band 3) *CRK***
