# Supplementary material for: Characterization of Leukemia-Inducing Genes Using a Proto-Oncogene/Homeobox Gene Retroviral Human cDNA Library in a Mouse In Vivo Model
Source: PLoS One. 2015 Nov 25;10(11):e0143240. doi: 10.1371/journal.pone.0143240 (PMC4659616; doi:10.1371/journal.pone.0143240)
Supplement: S4 Table — (DOCX) [file pone.0143240.s010.docx]

**S4 Table. Result of Next Generation Sequencing (NGS)** :

|  |  | **Retroviral cDNA Library (Plasmid Mix)** | **B6 BM Cells (Retrovirus-Transduced)** |
| --- | --- | --- | --- |
| **Gene_symbol** | **Description** | **FPKM** | **FPKM** |
| BMI1 | BMI1 polycomb ring finger oncogene | 7664.307834 | 4117.854753 |
| CDX1 | caudal type homeobox 1 | 772.550132 | 1005.629047 |
| CLDN1 | claudin 1 | 12400.23114 | 6731.043178 |
| CRK | v-crk avian sarcoma virus CT10 oncogene homolog | 9162.660949 | 12947.26627 |
| CRKL | v-crk avian sarcoma virus CT10 oncogene homolog-like | 1265.084651 | 911.198045 |
| CRX | cone-rod homeobox | 969.25261 | 876.156032 |
| DEK | DEK oncogene | 3858.844526 | 1274.174628 |
| DLX1 | distal-less homeobox 1 | 5942.911605 | 5502.072457 |
| DLX2 | distal-less homeobox 2 | 605.100361 | 677.171654 |
| DLX3 | distal-less homeobox 3 | 1423.105702 | 1130.251862 |
| DLX4 | distal-less homeobox 4 | 2546.425783 | 3242.540109 |
| DLX5 | distal-less homeobox 5 | 15811.41297 | 12772.03219 |
| ELK1 | ELK1, member of ETS oncogene family | 2872.561806 | 1073.561819 |
| ERBB3 | v-erb-b2 avian erythroblastic leukemia viral oncogene homolog 3 | 6770.65111 | 3463.05588 |
| ERG | v-ets avian erythroblastosis virus E26 oncogene homolog | 7174.673867 | 8577.727211 |
| ETAA1 | Ewing tumor-associated antigen 1 | 3011.38203 | 2809.82564 |
| ETS1 | v-ets avian erythroblastosis virus E26 oncogene homolog 1 | 6608.501944 | 3306.028672 |
| ETS2 | v-ets avian erythroblastosis virus E26 oncogene homolog 2 | 2058.962834 | 532.247992 |
| ETV6 | ets variant 6 | 2332.950307 | 1992.017823 |
| ETV7 | ets variant 7 | 2694.130569 | 1862.228909 |
| FES | feline sarcoma oncogene | 7732.73658 | 5144.118496 |
| FEV | FEV (ETS oncogene family) | 8069.205817 | 10290.58295 |
| FGFR1OP | FGFR1 oncogene partner | 6110.035509 | 2544.097782 |
| FGFR1OP2 | FGFR1 oncogene partner 2 | 10039.28954 | 7115.116396 |
| FGR | feline Gardner-Rasheed sarcoma viral oncogene homolog | 8819.096913 | 7100.82016 |
| FOS | FBJ murine osteosarcoma viral oncogene homolog | 3420.713897 | 1719.620168 |
| FOSB | FBJ murine osteosarcoma viral oncogene homolog B | 1.294732 | 0 |
| FYN | FYN oncogene related to SRC, FGR, YES | 1725.915393 | 1283.067384 |
| GL1 | glioma-associated oncogene homolog 1 (zinc finger protein), mRNA, complete | 11098.40512 | 7710.180722 |
| HHEX | hematopoietically expressed homeobox | 1532.406628 | 1733.401176 |
| HLX1 | Homo sapiens H2.0-like homeobox, mRNA, complete cds | 9112.067391 | 8930.387044 |
| HOXA1 | homeobox A1 | 896.122403 | 870.907763 |
| HOXA10 | homeobox A10 | 1415.121803 | 731.292855 |
| HOXA11 | homeobox A11 | 118.099963 | 58.016194 |
| HOXA3 | homeobox A3 | 576.69492 | 152.821891 |
| HOXA5 | homeobox A5 | 6318.885344 | 7392.158414 |
| HOXA6 | homeobox A6 | 10024.35924 | 4504.238006 |
| HOXA9 | homeobox A9 | 11271.12169 | 12585.39696 |
| HOXB1 | homeobox B1 | 6771.624339 | 2059.195241 |
| HOXB13 | homeobox B13 | 718.957601 | 551.794419 |
| HOXB2 | homeobox B2 | 294.951799 | 196.310062 |
| HOXB4 | homeobox B4 | 7140.94758 | 5593.302361 |
| HOXB5 | homeobox B5 | 1307.092615 | 997.021613 |

|  |  | **Retroviral cDNA Library (Plasmid Mix)** | **B6 BM Cells (Retrovirus-Transduced)** |
| --- | --- | --- | --- |
| **Gene_symbol** | **Description** | **FPKM** | **FPKM** |
| HOXB6 | homeobox B6 | 3890.941214 | 5980.172726 |
| HOXB7 | homeobox B7 | 8383.676962 | 10282.8784 |
| HOXB9 | homeobox B9 | 3951.79378 | 4943.717978 |
| HOXC10 | homeobox C10 | 3010.760599 | 1925.758047 |
| HOXC11 | homeobox C11 | 1929.384023 | 1970.790765 |
| HOXC13 | homeobox C13 | 10811.61048 | 7736.903992 |
| HOXC8 | homeobox C8 | 3990.641787 | 2035.239238 |
| HOXC9 | homeobox C9 | 8911.932302 | 6246.907122 |
| HOXD1 | homeobox D1 | 1751.482252 | 1480.201883 |
| HOXD10 | homeobox D10 | 0.833275 | 3.153327 |
| HOXD12 | homeobox D12 | 4420.502941 | 3330.255056 |
| HOXD3 | homeobox D3 | 2467.985318 | 1217.59239 |
| HOXD4 | homeobox D4 | 4568.940981 | 4718.420705 |
| HOXD8 | homeobox D8 | 0 | 1.354998 |
| HOXD9 | homeobox D9 | 3360.793646 | 2193.754713 |
| JUN | jun proto-oncogene | 1370.075227 | 1249.906516 |
| JUNB | jun B proto-oncogene | 4045.621201 | 3006.59361 |
| KRAS2 | v-Ki-ras2 Kirsten rat sarcoma viral oncogene homolog | 4410.002931 | 2110.092611 |
| LCN2 | lipocalin 2 | 18938.24067 | 19377.38956 |
| LHX1 | LIM homeobox 1 | 0 | 0 |
| LHX2 | LIM homeobox 2 | 6390.674237 | 3063.714412 |
| LHX4 | LIM homeobox 4 | 4096.946973 | 2016.607102 |
| LHX5 | LIM homeobox 5 | 3878.028392 | 3199.185824 |
| LHX6 | LIM homeobox 6 | 9100.845632 | 5665.84491 |
| LHX8 | LIM homeobox 8 | 6678.270934 | 8510.622207 |
| LHX9 | LIM homeobox 9 | 1009.02869 | 709.400009 |
| LYN | v-yes-1 Yamaguchi sarcoma viral related oncogene homolog | 0 | 0 |
| MAFB | v-maf avian musculoaponeurotic fibrosarcoma oncogene homolog B | 520.702139 | 528.813851 |
| MAFF | v-maf avian musculoaponeurotic fibrosarcoma oncogene homolog F | 6110.807218 | 4309.629254 |
| MAFG | v-maf avian musculoaponeurotic fibrosarcoma oncogene homolog G | 839.4332 | 1532.063283 |
| MAS1 | MAS1 oncogene | 3090.998275 | 4421.092071 |
| MAS1L | MAS1 oncogene-like | 3867.309081 | 4458.344514 |
| MEIS1 | Meis homeobox 1 | 691.364979 | 213.901916 |
| MEIS2 | Meis homeobox 2 | 0 | 0 |
| MEOX2 | mesenchyme homeobox 2 | 5346.531595 | 4057.615758 |
| MOS | v-mos Moloney murine sarcoma viral oncogene homolog | 3291.063921 | 2333.092861 |
| MSH2 | mutS homolog 2 | 4107.003961 | 3550.027035 |
| MSX2 | msh homeobox 2 | 2930.019904 | 2940.927901 |
| MYB | v-myb avian myeloblastosis viral oncogene homolog | 0 | 0 |
| MYBL1 | myb-related protein A isoform 3 | 8232.275194 | 5119.282618 |
| MYBL2 | v-myb avian myeloblastosis viral oncogene homolog-like 2 | 722.426891 | 534.037182 |
| MYC | v-myc avian myelocytomatosis viral oncogene homolog | 1716.73757 | 951.537336 |
| MYCL1 | v-myc myelocytomatosis viral oncogene homolog 1, lung carcinoma derived (avian) | 1188.826921 | 624.404911 |
| MYCNOS | MYCN opposite strand | 4566.535127 | 6543.988175 |
| NRAS | Homo sapiens neuroblastoma RAS viral (v-ras) oncogene homolog | 6143.568378 | 2186.406009 |

|  |  | **Retroviral cDNA Library (Plasmid Mix)** | **B6 BM Cells (Retrovirus-Transduced)** |
| --- | --- | --- | --- |
| **Gene_symbol** | **Description** | **FPKM** | **FPKM** |
| PBX3 | pre-B-cell leukemia homeobox 3 | 6602.692053 | 4992.070271 |
| PBX4 | pre-B-cell leukemia homeobox 4 | 657.094877 | 909.926035 |
| PBXIP1 | pre-B-cell leukemia homeobox interacting protein 1 | 2501.092748 | 3062.059224 |
| PDGFB | platelet-derived growth factor beta polypeptide | 986.926473 | 1365.578271 |
| PIM1 | pim-1 oncogene | 3837.164446 | 2865.01519 |
| PIM2 | pim-2 oncogene | 1563.268955 | 1433.184552 |
| PIM3 | pim-3 oncogene | 9029.107201 | 9901.702659 |
| POU2F2 | POU class 2 homeobox 2 | 4677.022911 | 778.290883 |
| POU2F3 | POU class 2 homeobox 3 | 8180.550201 | 9913.958164 |
| POU4F3 | POU class 4 homeobox 3 | 219.044819 | 311.072962 |
| POU5F1 | POU class 5 homeobox 1 | 4418.090216 | 5190.626509 |
| POU6F1 | POU class 6 homeobox 1 | 6610.009591 | 8008.419374 |
| PROX1 | prospero homeobox 1 | 470.374073 | 319.358706 |
| PTTG1 | pituitary tumor-transforming 1 | 0 | 0 |
| RAB10 | RAB10, member RAS oncogene family | 4038.049891 | 4316.86232 |
| RAB11A | RAB11A, member RAS oncogene family | 1782.382125 | 1763.656012 |
| RAB13 | RAB13, member RAS oncogene family | 14598.28262 | 15983.84839 |
| RAB14 | RAB14, member RAS oncogene family | 332.982875 | 298.421437 |
| RAB17 | RAB17, member RAS oncogene family | 7051.711465 | 7859.050924 |
| RAB18 | RAB18, member RAS oncogene family | 2191.721193 | 1194.372871 |
| RAB1A | RAB1A, member RAS oncogene family | 1567.45802 | 1746.243966 |
| RAB1B | RAB1B, member RAS oncogene family | 2880.024267 | 2862.41688 |
| RAB2A | RAB2A, member RAS oncogene family | 10092.18846 | 9902.831159 |
| RAB22A | RAB22A, member RAS oncogene family | 662.990599 | 651.396781 |
| RAB23 | RAB23, member RAS oncogene family | 4225.184857 | 3648.352347 |
| RAB25 | RAB25, member RAS oncogene family | 5504.656834 | 7742.919297 |
| RAB27B | RAB27B, member RAS oncogene family | 2044.846203 | 1952.149469 |
| RAB2A | RAB2A, member RAS oncogene family | 2906.212504 | 3098.554481 |
| RAB2B | RAB2B, member RAS oncogene family | 4976.802793 | 4825.55972 |
| RAB30 | RAB30, member RAS oncogene family | 1916.78008 | 2043.913581 |
| RAB31 | RAB31, member RAS oncogene family | 5837.7534 | 5696.009269 |
| RAB33A | RAB33A, member RAS oncogene family | 25180.35022 | 23108.08963 |
| RAB34 | RAB34, member RAS oncogene family | 2568.117005 | 4583.66546 |
| RAB35 | RAB35, member RAS oncogene family | 3694.651468 | 4606.603 |
| RAB37 | RAB37, member RAS oncogene family | 0 | 0 |
| RAB38 | RAB38, member RAS oncogene family | 9130.811641 | 11605.83397 |
| RAB39 | RAB39, member RAS oncogene family | 9910.790146 | 11400.42339 |
| RAB3A | RAB3A, member RAS oncogene family | 18067.02376 | 17527.86839 |
| RAB3B | RAB3B, member RAS oncogene family | 599.515532 | 685.447132 |
| RAB3C | RAB3C, member RAS oncogene family | 424.88094 | 507.236396 |
| RAB3D | RAB3D, member RAS oncogene family | 2301.539737 | 2271.058814 |
| RAB40B | RAB40B, member RAS oncogene family | 428.525343 | 384.299419 |
| RAB4A | RAB4A, member RAS oncogene family | 11948.99859 | 7407.179913 |
| RAB5A | RAB5A, member RAS oncogene family | 5106.842285 | 3122.383212 |
| RAB5C | RAB5C, member RAS oncogene family | 2131.202019 | 1159.027891 |
| RAB6A | RAB6A, member RAS oncogene family | 411.599925 | 561.599067 |
| RAB6B | RAB6B, member RAS oncogene family | 2568.360808 | 2420.909916 |
| RAB7B | RAB7B, member RAS oncogene family | 0 | 0 |
| RAB7L1 | RAB7, member RAS oncogene family-like 1 | 1430.194331 | 1508.377265 |
| RAB8A | RAB8A, member RAS oncogene family | 6207.860504 | 6345.624967 |
| RAB8B | RAB8B, member RAS oncogene family | 4765.506518 | 4873.788178 |

|  |  | **Retroviral cDNA Library (Plasmid Mix)** | **B6 BM Cells (Retrovirus-Transduced)** |
| --- | --- | --- | --- |
| **Gene_symbol** | **Description** | **FPKM** | **FPKM** |
| RAB9A | RAB9A, member RAS oncogene family | 26609.62466 | 25668.18022 |
| RABL2B | RAB, member of RAS oncogene family-like 2B | 11548.76871 | 11380.58387 |
| RABL3 | RAB, member of RAS oncogene family-like 3 | 5862.364466 | 5583.533056 |
| RABL4 | RABL4, member of RAS oncogene family-like 4 | 0 | 0 |
| RABL5 | RAB, member RAS oncogene family-like 5 | 9249.497364 | 12560.33892 |
| RAF1 | v-raf-1 murine leukemia viral oncogene homolog 1 | 1749.739174 | 2771.207084 |
| RALA | v-ral simian leukemia viral oncogene homolog A (ras related) | 7711.652529 | 8135.266489 |
| RALB | v-ral simian leukemia viral oncogene homolog B | 2857.210628 | 1993.547688 |
| RAN | RAN, member RAS oncogene family | 7539.048424 | 7869.698849 |
| RAP1A | RAP1A, member of RAS oncogene family | 5699.75496 | 7606.59289 |
| RAP1B | RAP1B, member of RAS oncogene family | 11398.37739 | 12230.93423 |
| RAP2B | RAP2B, member of RAS oncogene family | 1305.202922 | 1973.134077 |
| RAP2C | RAP2C, member of RAS oncogene family | 2715.77101 | 3150.823509 |
| RELB | v-rel avian reticuloendotheliosis viral oncogene homolog B | 1347.727117 | 222.527075 |
| RET | ret proto-oncogene | 0 | 0 |
| RRAS | related RAS viral (r-ras) oncogene homolog | 10409.96841 | 6084.288691 |
| RRAS2 | related RAS viral (r-ras) oncogene homolog 2 | 4082.317727 | 4411.555927 |
| SET | SET nuclear oncogene | 2829.077206 | 2170.97331 |
| SIX1 | SIX homeobox 1 | 1715.287191 | 1661.210702 |
| SPI1 | spleen focus forming virus (SFFV) proviral integration oncogene | 7305.695265 | 6256.988752 |
| SSPN | sarcospan | 1702.003521 | 1299.502856 |
| TACSTD1 | tumor-associated calcium signal transducer 2 | 9011.067389 | 6608.394104 |
| TACSTD2 | tumor-associated calcium signal transducer 2 | 14420.71513 | 8160.676833 |
| TGIF1 | TGFB-induced factor homeobox 1 | 11933.07009 | 12279.57859 |
| TGIF2 | TGFB-induced factor homeobox 2 | 2077.892435 | 2645.019207 |
| THRA | thyroid hormone receptor, alpha | 10807.32125 | 2569.977726 |
| THRB | thyroid hormone receptor, beta | 0 | 0 |
| TIMP1 | TIMP metallopeptidase inhibitor 1 | 2290.70173 | 3110.900288 |
| TLX1 | T-cell leukemia homeobox 1 | 11090.09623 | 5894.096811 |
| TLX2 | T-cell leukemia homeobox 2 | 7818.349166 | 3190.763471 |
| TLX3 | T-cell leukemia homeobox 3 | 1772.059301 | 1422.923965 |
| TMEM205 | transmembrane protein 205 | 2212.197335 | 1210.567778 |
| USP4 | ubiquitin specific peptidase 4 (proto-oncogene) | 1150.788054 | 1772.059265 |
| VAX2 | ventral anterior homeobox 2 | 4647.479691 | 2211.96672 |
| WT1 | Wilms tumor 1 | 5551.046264 | 4412.096812 |
| WTAP | Wilms tumor 1 associated protein | 6620.064831 | 5510.825027 |
| YES1 | v-yes-1 Yamaguchi sarcoma viral oncogene homolog 1 | 5642.569056 | 1096.763099 |

NGS was performed by Macrogen Inc., Seoul, Korea using Illumina Hiseq 2000 platform. The whole mixture of PCR products amplified from plasmid mixture or from the genomic DNA of bone marrow cells infected with retroviral cDNA library were analyzed through transcriptome analysis tools, and the data was presented as FPKM (Fragments Per Kilobase of transcript per Million mapped reads)

Data available from the Dryad Digital Repository: http://dx.doi.org/10.5061/dryad.2h0g7
